# Assessing Dose-Exposure–Response Relationships of Miltefosine in Adults and Children using Physiologically-Based Pharmacokinetic Modeling Approach

**DOI:** 10.1007/s11095-023-03610-0

**Published:** 2023-10-10

**Authors:** Shadrack J. Madu, Ke Wang, Siri Kalyan Chirumamilla, David B. Turner, Patrick G. Steel, Mingzhong Li

**Affiliations:** 1 School of Pharmacy, De Montfort University, Leicester, LE1 9BH UK; 2 Certara UK Limited, Simcyp Division, Sheffield, S1 2BJ UK; 3 Department of Chemistry, Durham University, Durham, DH1 3LE UK

**Keywords:** dose-exposure–response relationships, miltefosine, PBPK modelling, pharmacokinetics

## Abstract

**Objectives:**

Miltefosine is the first and only oral medication to be successfully utilized as an antileishmanial agent. However, the drug is associated with differences in exposure patterns and cure rates among different population groups e.g. ethnicity and age (i.e., children v adults) in clinical trials. In this work, mechanistic population physiologically-based pharmacokinetic (PBPK) models have been developed to study the dose-exposure–response relationship of miltefosine in *in silico* clinical trials and evaluate the differences in population groups, particularly children and adults.

**Methods:**

The Simcyp population pharmacokinetics platform was employed to predict miltefosine exposure in plasma and peripheral blood mononuclear cells (PBMCs) in a virtual population under different dosing regimens. The cure rate of a simulation was based on the percentage of number of the individual virtual subjects with AUC_d0-28_ > 535 µg⋅day/mL in the virtual population.

**Results:**

It is shown that both adult and paediatric PBPK models of miltefosine can be developed to predict the PK data of the clinical trials accurately. There was no significant difference in the predicted dose-exposure–response of the miltefosine treatment for different simulated ethnicities under the same dose regime and the dose-selection strategies determined the clinical outcome of the miltefosine treatment. A lower cure rate of the miltefosine treatment in paediatrics was predicted because a lower exposure of miltefosine was simulated in virtual paediatric in comparison with adult virtual populations when they received the same dose of the treatment.

**Conclusions:**

The mechanistic PBPK model suggested that the higher fraction of unbound miltefosine in plasma was responsible for a higher probability of failure in paediatrics because of the difference in the distribution of plasma proteins between adults and paediatrics. The developed PBPK models could be used to determine an optimal miltefosine dose regime in future clinical trials.

**Graphical Abstract:**

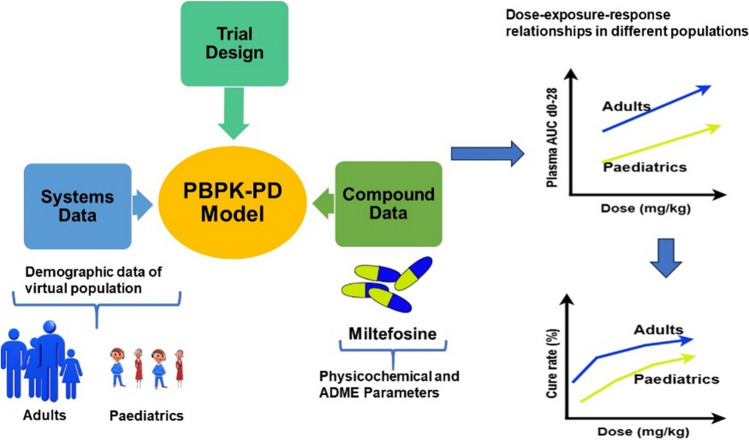

**Supplementary Information:**

The online version contains supplementary material available at 10.1007/s11095-023-03610-0.

## Introduction

Leishmaniasis is a neglected tropical disease caused by protozoan *Leishmania* parasites transmitted during blood meal by infected female sandflies or, to a lesser extent, through accidental contact with infected human blood [1]. Human leishmanial infections may manifest as cutaneous leishmaniasis, mucocutaneous leishmaniasis, or visceral leishmaniasis. Cutaneous leishmaniasis is the most common form, a group of diseases with a varied spectrum of clinical manifestations, whilst visceral leishmaniasis is the most severe form, in which the parasites have migrated to vital organs [2]. Leishmaniasis mainly affects the poorer populations, especially in the subtropical and tropical regions of the globe, with a negative socioeconomic impact on the infected individuals and their community. According to the World Health Organisation report in 2022 (https://www.who.int/news-room/fact-sheets/detail/leishmaniasis), an estimated 700,000 to 1 million new cases occur annually, causing significant morbidity and mortality in Africa, Asia, and Latin America.

**Fig. 1:**
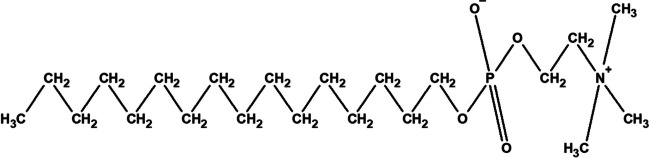
Molecular structure of miltefosine.

The current drug treatments for leishmaniasis are limited and poorly tolerated. They include pentavalent antimonials, paromomycin, pentamidine, amphotericin B, and miltefosine [3]. Among these, miltefosine, an alkylphosphocholine drug (C_21_H_46_NO_4_P in Fig. 1), is the only available oral drug for treating leishmaniasis in the World Health Organization list of essential medicines [2, 4]. Over the last two decades, clinical trials of miltefosine have been conducted in both cutaneous and visceral leishmaniasis patient populations from the Indian subcontinent [5–8], East Africa (i.e., Kenya and Sudan) [9], Colombia [10], and Europe [11]. For adults, the optimum monotherapy for miltefosine is 2.5 mg/kg body weight daily for 28 days, leading to higher cure rates of 86% for Eastern African patients and 97% for Indian patients at six-month follow-ups [12]. In contrast, a significantly higher probability of failure was found for children treated with the same linear dosing of 2.5 mg/kg/day of miltefosine [9, 13]. For example, the cure rate in Nepal and eastern African paediatric patients was just 59% at six month’s follow-up [12]. Clinical pharmacokinetic analyses have shown that lower miltefosine exposure is the main cause of the relatively poor efficacy rates of miltefosine in paediatrics compared with adult patients. Based on a two-compartment or three-compartment pharmacokinetic (PK) modelling approach using the clinical data fitting, a significantly higher dosing of miltefosine in children based on fat-free mass (FFM) up to 3.9 mg/kg was proposed, aiming to produce a profile of drug exposure similar to that observed in adults [14–17]. Subsequently, this proposed dosing regimen was tested in East African children with visceral leishmaniasis, resulting in a 90% cure rate at six months follow-up [14, 18]. However, a mechanistic understanding of the changes to miltefosine effects in children and adults or different ethnic populations related to pharmacokinetics and pharmacodynamics is lacking.

Physiologically-based pharmacokinetic (PBPK) modelling is a well-established approach for simulating the *in vivo* mechanisms of absorption, distribution, metabolism, and excretion (ADME) of a drug candidate following administration in individuals under various physiological conditions [19]. PBPK models integrate anatomical and physiological parameters of animals or humans, physicochemical properties of drug substances, and formulation properties of drug products to predict and simulate the PK parameters of drugs in virtual populations following administration of similar doses, providing insights into the issue of variability of PK profiles in special population groups [20]. Currently, several *in silico* PBPK modelling tools are available, such as GastroPlus, Simcyp, SimBiology, PhysPK and PK Sim. They have been applied to all stages of drug development, ranging from lead optimization in the drug discovery phase through clinical candidate selection and formulation development to exploring drug-drug interactions to support the approval process for regulatory requirements [21–30]. Thus, population-based PBPK modelling could be a useful approach to predict the mean and population variability of the *in vivo* behaviour of miltefosine to design effective and safe dosing treatments according to the ethnic, disease, paediatric, or adult population of interest.

The aim of this study was to develop a mechanistic population-based PBPK model to predict the PK profiles of miltefosine in *in silico* clinical trials and to correlate and understand the different efficacy of the treatment in different population groups, particularly between children and adults. This work employed the Simcyp population-PBPK platform (https://www.certara.com/software/simcyp-pbpk/) to predict miltefosine plasma concentrations in a virtual population under different dosing regimens. The cure rate of miltefosine treatment is related to systemic drug exposure in plasma and also depends on its concentration in the host cells because leishmania parasites are intracellular pathogens [10]. Therefore, the prediction of the miltefosine distribution in peripheral blood mononuclear cells (PBMCs) in a virtual population was implemented within the developed PBPK model using the PD (pharmacodynamics) model facility in the software. Finally, the dose-exposure–response relationships of miltefosine in adults and children were assessed by the developed PBPK models with a PK target of AUC_d0-28_ > 535 µg⋅day/mL in plasma [31].

## Materials and Methods

### Patient Populations and Pharmacokinetic data

The patient demographics and pharmacokinetic data were obtained from the clinical trials and their corresponding pharmacokinetic modelling studies [9, 10, 15–18, 31, 32].

The adult studies included individuals of European, Nepalese, Afro-Colombian and East African origins. The Europeans were 31 Dutch military personnel with cutaneous leishmaniasis who were treated with 150 mg/day of miltefosine for 28 days [32]. Nepalese patients with visceral leishmaniasis were treated with 100 mg/day of miltefosine for 28 days [15], whilst East African adult patients with visceral leishmaniasis and Afro-Colombian adult patients with cutaneous leishmaniasis were treated with the 2.5 mg/kg/day regimen for 28 days [9, 10, 16, 31].

Afro-Colombian children with cutaneous leishmaniasis received miltefosine at a nominal dose of 2.5 mg/kg/day for 28 days [10, 31]. Two groups of East African children with visceral leishmaniasis received miltefosine at a nominal dose of 2.5 mg/kg/day or the allometric dosing regimen of between 2.7 and 3.9 mg/kg/day based on FFM for 28 days [17, 18].

### Population-based PBPK Modelling and Simulations

A PBPK model for miltefosine in human was developed using the Simcyp Human Simulator (Version 22, Certara UK Limited, Sheffield, UK). There are three key elements of a mechanistic population PBPK model: the drug characteristics, virtual human populations (systems data), and the trial design.

#### Drug-Specific and System-Specific Components

The drug characteristics include its physicochemical properties, which remain unaltered in adult or paediatric models, and its properties to describe the absorption and elimination processes, which could be modified from adults to children or different ethnic groups.

i) Physicochemical and blood binding properties
  Miltefosine’s physicochemical properties were obtained from literature as shown in Table I. Miltefosine is a monoprotic acid with a molar mass of 407.576 g/mol and its pKa, logP_O:W_ (octanol:water partition coefficient) values are 2 and 3.7 respectively [33]. The miltefosine concentration in whole blood is 86% of that in plasma (i.e., B/P ratio of 0.86) [34]. Miltefosine is also characterised by a high plasma protein binding ranging from 96 to 98%, hence, the fraction unbound in plasma (f_u_) was set to be 0.02 [12].
ii) Absorption phase
  Miltefosine absorption was described by the Advanced Dissolution, Absorption, and Metabolism (ADAM) model of the Simcyp Simulator. The ADAM model has nine anatomically defined segments from the stomach through the intestine to the colon. It can be used to model food effects and various formulation effects [35]. The required absorption input parameters for the miltefosine ADAM model are shown in Table I. The fraction of miltefosine unbound in the enterocytes (f_uGut_), which defines the fraction of drug entering the enterocytes available for first-pass gut metabolism, was set to the default value of 1. The value of f_uGut_ can have a big impact on first pass metabolism and thus prediction of plasma drug concentration. Measured values are not available and, therefore, an automated sensitivity analysis (ASA) was performed to investigate the impact of the uncertainty of f_uGut_ on the predictions (see Results section).
  The regional effective gut wall permeabilities were predicted by the built-in MechPeff Model [36]. The intrinsic membrane permeability (P_trans,0_) was predicted from logP_o:w_ with the built-in Method 2 logP_ow_-P_trans,0_ correlation function. The effective regional permeabilities are given in Table I. The Absorption and Basolateral Permeability rate scalars were left at default values of 1; *i.e.,* they have no influence on the model.
  As miltefosine is freely soluble in an aqueous medium (≥ 2.5 mg/mL), the type of formulation, such as tablet or solution, is not expected to affect its oral absorption [33]. Therefore, the ADAM model with “solution” as the formulation type was selected.
iii) Distribution
  A full PBPK model of distribution was employed for miltefosine in the Simcyp Simulator in order to simulate drug concentrations in various organ compartments (i.e., blood/plasma, adipose, bone, brain, gut, heart, kidneys, liver, lungs, muscle, pancreas, skin, and spleen). It can also consider interindividual variability (e.g., specific age, sex, weight, and height) in predicting tissue volumes. The volume of distribution at steady state (V_ss_) was reported in the literature to be 0.96 L/Kg [37]. Method 2 (based on Rodgers and Rowland) [38–40] was selected to predict Vss and the tissue-plasma partition coefficients (Kps) and the K_p_ Scalar adjusted to 17.6 to reproduce the observed Vss. An ASA on K_p_ scalar was performed to refine the PBPK model, detailed in the Results section.
iv) Elimination
  It has been shown that the main metabolic pathway of miltefosine is mediated by phospholipase D [41]. As esterase enzymes hydrolyse ester, amide, and thioester bonds, phospholipase D hydrolyses miltefosine to choline, choline-containing metabolites, and hexadecanol [42]. Miltefosine is not a substrate of cytochrome P450 metabolic enzymes and only 0.2% of the administered dose is eliminated in the urine at day 23 of a 28-day treatment regimen [41, 43]. Therefore, phospholipase D input as an esterase was employed as the only elimination pathway in the Simcyp Simulator. The input parameter of CL_int_ (intrinsic clearance of miltefosine) or V_max_/K_m_ (V_max_ is the maximum rate of the enzymatic reaction; K_m_ is the concentration of the drug which permits the enzyme to achieve half V_max_) is needed for phospholipase D kinetics. As none of these parameters are/were available in the literature, CL_int_ was back-calculated from the oral clearance, CL_po,_ based on the net intrinsic hepatic clearance [${\mathrm{CL}}_{\mathrm{int},\mathrm{ H}} (\mathrm{L}/\mathrm{h})]$ using Eq. (1) as [44].
  1 $${{\mathrm{CL}}_{\mathrm{int}}=\mathrm{CL}}_{\mathrm{int},\mathrm{ H}} (\mathrm{L}/\mathrm{h})=\frac{{\mathrm{CL}}_{\mathrm{po}}*{\mathrm{f}}_{\mathrm{uGut}}*{\mathrm{f}}_{\mathrm{a}}-{\mathrm{CL}}_{\mathrm{R}}}{{\mathrm{fu}}_{\mathrm{B}} \left(1+\frac{{\mathrm{CL}}_{\mathrm{R}}}{{\mathrm{Q}}_{\mathrm{H},\mathrm{B}}}\right)}$$
  where ${\mathrm{fu}}_{\mathrm{B}}$ is the fraction of drug unbound in the blood, which can be calculated as ${\mathrm{fu}}_{\mathrm{B}}=\frac{{\mathrm{f}}_{\mathrm{u}}}{\mathrm{B}/\mathrm{P}}=\frac{0.02}{0.86}=0.023$; ${\mathrm{CL}}_{\mathrm{R}}$ is the renal clearance, which was set as zero discussed above; ${\mathrm{f}}_{\mathrm{a}}$ is the fraction of the drug absorbed from the gut, which was set as 1, based on the assumption of complete absorption of miltefosine from the gut; ${\mathrm{Q}}_{\mathrm{H},\mathrm{B}}$ is the hepatic blood flow, which was set as 88.887 L/h predicted by the Simcyp simulator reverse translational tool. The oral clearance was obtained as ${\mathrm{CL}}_{\mathrm{po}}=4.62\mathrm{ L}/\mathrm{day }=0.1925\mathrm{ L}/\mathrm{h}$ from the literature [31] and f_uGut_ was set to an initial value of 1 as noted above. Therefore, the net intrinsic hepatic clearance was ${\mathrm{CL}}_{\mathrm{int},\mathrm{ H}} (\mathrm{L}/\mathrm{h})=8.2775$. Unit change is also required for the CL_int_ in the software as
  2 $${\mathrm{CL}}_{\mathrm{int},\mathrm{ H}} (\mathrm{uL}/\mathrm{min}/\mathrm{mg})=\frac{{\mathrm{CL}}_{\mathrm{int},\mathrm{ H}} (\mathrm{L}/\mathrm{h})\;*\;1000\;*\;1000}{\mathrm{Average\; liver \;wt}\;*\;\mathrm{milligram\; of\; mic\; protein\; per\; gram \;of\; liver}*60}$$
  where average liver weight is $1.6\times {10}^{3}$ g while milligram of microsomal protein per gram of liver is 36.544 mg, was predicted with the Simcyp Reverse Translational Tool (Retrograde model). The input parameters were Sim-NEurCaucasians with age from 21 to 51 years and proportion of females 0.55, which are based on the clinical trial data. Therefore, the initial ${\mathrm{CL}}_{\mathrm{int},\mathrm{ H}} (\mathrm{uL}/\mathrm{min}/\mathrm{mg})$ was 2.36 which was calculated manually. Furthermore, an ASA of ${\mathrm{CL}}_{\mathrm{int},\mathrm{ H}} (\mathrm{uL}/\mathrm{min}/\mathrm{mg})$ was performed to refine the PBPK model (see Results section).
  The fraction of the unbound drug in the *in vitro* hepatocyte incubation fu,_inc_ was left at a default value of 1 as the CL_int_ was backcalculated. The tissue activity scalars of the liver, intestine and kidney were left at 1 as phospholipase D is available in all body tissues.
v) Miltefosine concentration profile in PBMCs
  The intracellular concentration–time profile of miltefosine was predicted using the PD basic unit within Simcyp Simulator [45]. As the intracellular PBMC miltefosine steady-state concentrations were found to be around twofold higher than plasma concentrations [31], a linear response model in Eq. (3) was employed to predict the intracellular PBMC miltefosine concentration with the miltefosine plasma concentration as the model input, detailed as
  3 $${C}_{IC}={E}_{0}+\alpha .{C}_{PL}$$
  where, ${C}_{IC}$ is the predicted intracellular concentration of miltefosine; ${E}_{0}$ is the baseline effect which was set to 0; ${C}_{PL}$ is the predicted total plasma concentration of miltefosine; α is the activation constant enhancement factor, which is the slope of the model with an initial default value of one (Table I). An ASA on α was carried out to optimise the prediction of the intracellular concentration of miltefosine, detailed in the Results section.

**Table I:** Input Parameters for Miltefosine PBPK Model Simulations in Simcyp Human Simulator

Parameter	Initial value	Reference/Comments	Optimised final value for adults (children)
Physicochemical properties and blood binding			
Molecular weight (g/mol)	407.57		407.57
logP_o:w_	3.7	[33]	3.7
Compound type	Monoprotic acid		
pKa	2	[33, 41]	2
B/P	0.86	[34]	0.86
f_u_	0.02	[12]	0.02 (0.04)
Absorption Phase			
Model	ADAM		
f_uGut_	1	Default value of 1. ASA indicated the model is not sensitive to its value	1
Permeability Model	Mechpeff Model		
Ptrans,0 (10^−6^ cm/s)	1.5255 × 10^5^	Predicted from logP_o:w_	1.5255 × 10^5^
P_eff,man_ (10^−4^ cm/s) (Duodenum)	0.93	Predicted	0.93
P_eff,man_ (10^−4^ cm/s) (Jejunum I)	1.00	Predicted	1.00
P_eff,man_ (10^−4^ cm/s) (Jejunum II)	0.76	Predicted	0.76
P_eff,man_ (10^−4^ cm/s) (Ileum I)	0.32	Predicted	0.32
P_eff,man_ (10^−4^ cm/s) (Ileum II)	0.31	Predicted	0.31
P_eff,man_ (10^−4^ cm/s) (Ileum III)	0.31	Predicted	0.31
P_eff,man_ (10^−4^ cm/s) (Ileum IV)	0.30	Predicted	0.30
P_eff,man_ (10^−4^ cm/s) (Colon)	0.14	Predicted	0.14
Absorption Rate Scalar: Global	1	Default value	1
Basolateral and Permeability Scalars: Global	1	Default value	1
Formulation	Solution		
Distribution Phase			
Simulation Model	Full PBPK		
V_ss_ (L/kg)	0.96	An initial value of 0.96 was obtained from the literature [37]. Using ASA the Kp Scalar was optimised to 0.31	0.31
K_p_ Scalar	17.6	Value first adjusted to obtain the observed Vss. An ASA was conducted to obtain an optimised value of 4.92	4.92
Elimination phase			
Metabolic enzyme input	Phospholipase D (Added under esterases as user input)		
Tissue Activity Scalars (i.e., Liver, Intestine and Kidney)	1,1 and 1, respectively		1,1 and 1, respectively
CL_int_ (uL/min/mg)	2.36	An initial value (2.36) back-calculated [44]. ASA was performed to obtain an optimised value of 1.87	1.87 (1.18)
CL_R_ (L/h)	0	Renal excretion was neglected as only 0.2% of the administered dose was eliminated at day 23 of a 28-day treatment regimen [41, 43]	0
Pharmacodynamic Model	Linear Response Model		
α	1	The default value of one was set as the initial value then ASA was used to give an optimised value of 0.8	0.8
${E}_{0}$	0	Default value	0

*B/P* Blood-to-plasma partition ratio, *fu*: fraction of unbound drug in plasma, *ADAM model* Advanced, Dissolution, Absorption and Metabolism model, f_uGut_ Unbound fraction of drug in enterocytes, P_trans,0_ intrinsic membrane permeability, P_eff,man_ Human jejunum effective permeability, V_ss_ volume of distribution at steady state, K_p_ Scalar Tissue-plasma partition coefficient, CL_int_ intrinsic clearance, CL_R_ Renal Clearance, α the activation constant enhancement factor for bound receptor, E_0_ Baseline effect

#### Population and Trial Design

Several ethnic populations are available in the Simcyp Simulator, including adult populations (e.g., Chinese, Japanese, North European Caucasian, North American African American, North American Asian, North American Hispanic_Latino, and so on) and paediatric populations (e.g., Sim-Paediatric based on North European paediatrics, Chinese paediatrics, and Japanese paediatrics). This work used the PK data originating from an open-label clinical trial for adult Colombian patients with cutaneous leishmaniasis for the PBPK model development [10, 31]. Thus, the North American Hispanic_Latino database was employed to generate virtual subjects with similar demographic characteristics to the actual clinical trial populations. To assess ethnic differences, virtual African, European, and Nepalese populations generated by modification of the existing populations North American African American, North European Caucasian and North American Asian. Simulations were conducted and the predictivity of the PBPK models assessed against the clinical data.

As the drug-protein binding and clearance were the main age-dependent parameters available in the PBPK model, an ASA was performed on these two parameters to help create a more predictive model for children where the clinical data were available [46]. In the simulation, the virtual paediatric subjects were generated using the built-in Sim-Paediatric population. The clinical PK data used in the paediatric PBPK model development and validation were based on the clinical trials of Afro-Colombian children with cutaneous leishmaniasis and East Africa children with visceral leishmaniasis [10, 17, 18, 31].

Regarding trial design, the simulations were matched closely with those in the selected clinical trials based on the age, gender, weight and height.

### Assessment of PBPK Model Accuracy

The predictive accuracy of the PBPK models was assessed by both the fold error and cure rate.

1. Fold Error (FE)
  The fold error is defined as the ratio of the predicted PK values with the observed values obtained in the actual clinical trials.
  4 $$\mathrm{FE}=\frac{{\mathrm{X}}_{\mathrm{predicted}}}{{\mathrm{X}}_{\mathrm{observed}}}$$
  where ${\mathrm{X}}_{\mathrm{predicted}}$ represents the simulated mean value of the plasma (or intracellular) maximum concentration (C_max_), the time to reach the maximum concentration (T_max,_), or the area under the plasma concentration–time curve from days 0 to 28 (AUC_d0-28_) or days 0 to infinite AUC_d0-∞_. ${\mathrm{X}}_{\mathrm{observed}}$ is the observed mean value obtained in an actual clinical trial.
  If the FE values were within the range 0.5 to 2.0, it was concluded that the virtual clinical trial was successful. Additionally, if the FE values were in the range 0.8 to 1.25, it indicated the accuracy of the model prediction was excellent. Otherwise, the model was not successful [47].
2. Cure Rate (CR)
  Based on the PK-PD analyses, a PK target for cutaneous leishmaniasis was proposed where AUC_d0-28_ > 535 µg⋅day/mL, corresponding to more than 95% probability of a cure [31]. Therefore, the predicted CR is defined as
  5 $$\mathrm{CR}=\frac{{\mathrm{N}}_{{\mathrm{AUC}}_{\mathrm{d}0-28}> 535\mathrm{ \mu g}\cdot \mathrm{day}/\mathrm{mL}}}{{\mathrm{N}}_{\mathrm{total}}}\times 100\mathrm{\%}$$
  where ${\mathrm{N}}_{{\mathrm{AUC}}_{\mathrm{d}0-28}> 535\mathrm{ \mu g}\cdot \mathrm{day}/\mathrm{mL}}$ is the number of individual virtual subjects with AUC_d0-28_ > 535 µg⋅day/mL and ${\mathrm{N}}_{\mathrm{total}}$ is the total number of the virtual subjects in the trial simulation.

## Results

### Demographics and Pharmacokinetic Data Analyses

The miltefosine PK data obtained from different actual clinical trials and their corresponding pharmacokinetic modelling studies are summarized as:

1. Open-label pharmacokinetic clinical trial in children and adults with cutaneous leishmaniasis in Colombia [10, 31]
  In this study, 51 patients (29 children aged 2 to 12 years and 22 adults aged 21 to 51) were enrolled and received miltefosine at a normal dose of 2.5/kg/day for 28 days. Details of the demographic data of the patients are shown in Table S1. The PK data are available in both plasma and intracellular PBMCs, i.e., C_max_, T_max_, t_½_, AUC_d0-28_ and AUC_d0-∞_ in plasma and C_max_, T_max_, and AUC_d0-28_ in intracellular PBMCs.
2. Open-label randomized multicentre study in East Africa [9, 16]
  This was a phase II open-label, non-comparative randomized trial conducted in Kenya and Sudan. Three treatment regimens were evaluated, including a combination therapy of AmBiosome and Sodium Stibogluconate, a combination therapy of AmBiosome and miltefosine and miltefosine alone [9]. For the monotherapy of miltefosine, the participants included adults and paediatric subjects aged 7–41 years infected with visceral leishmaniasis. Miltefosine was administered at 2.5 mg/kg/day for 28 days, and patients followed up for 210 days. Pharmacokinetic parameters reported for adults are AUC_d0-28_ and AUC_d0-∞_ in plasma.
3. Comparative two clinical trials in children with visceral leishmaniasis in East Africa [17, 18]
  In this study, children from Kenya, Sudan and Uganda were treated at a normal dose of 2.5 mg/kg based on the linear weight-based dosing regimen or at daily doses of between 2.7 or 3.9 mg/kg based on the FFM allometric dosing regimen for 28 days [17]. Available PK data from these studies are C_max_, AUC_d0-7_, AUC_d0-28_, and AUC_d0-∞_.
4. Open-label, nonrandomized clinical trial in Europe [32]
  An extensive clinical trial of miltefosine involving 31 (3.2% female) Dutch military personnel aged between 23 to 29 years old who were infected with cutaneous leishmaniasis from Afghanistan. A daily dose of 50 mg three times daily (equivalent to 150 mg/day) was administered orally for 28 days. Patients were examined for about six months after discontinuation of treatment. The pharmacokinetic parameters reported include C_max_, T_max_, and t_½_.
5. Population Pharmacokinetic-Pharmacodynamic study of miltefosine in Nepal [15]
  This clinical trial was conducted in a Nepalese referral hospital involving 81 (38.2% female) confirmed visceral leishmaniasis patients aged between 2 to 65. Patients were treated with miltefosine according to the Nepalese National treatment guideline, adults (defined as ≥ 12 years of age) with a body weight of > 25 kg received 50 mg twice daily (total daily dose of 100 mg/day), adults weighing ≤ 25 kg received 50 mg once daily and children (2–11 years of age) received 2.5 mg/kg body weight /day rounded to 10 mg. Treatment was for 28 days, and follow-up visits were extended to 12 months after the completion of therapy. The pharmacokinetic parameters available are C_max_, AUC_d0-28_, and AUC_d0-∞_ in plasma.

The summary of the PK data of the clinical trials is shown in Table S1 in the supporting materials.

### Miltefosine PBPK Model for Adults

#### Miltefosine PBPK Model Development

**Fig. 2:**
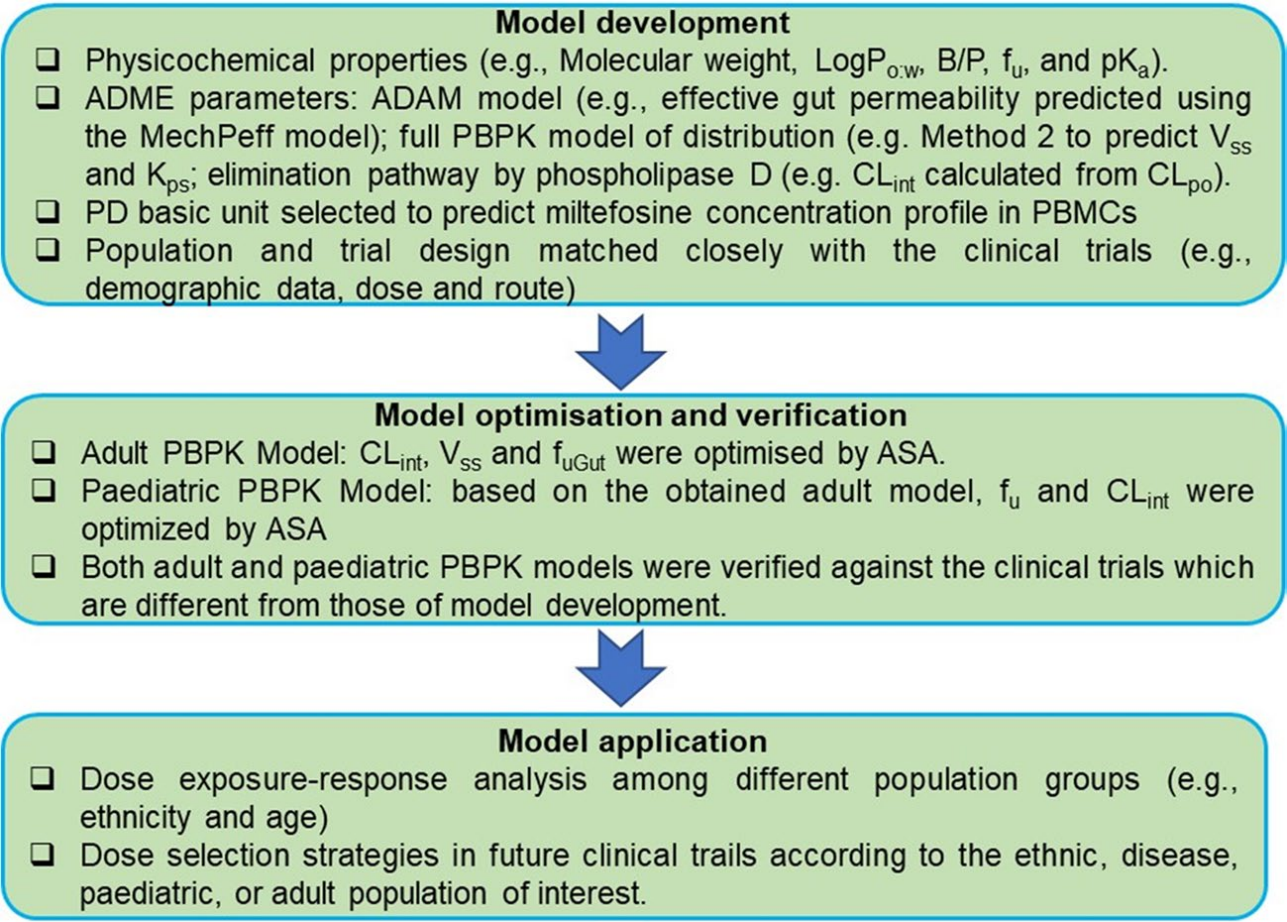
Flowchart for miltefosine PBPK model development.

The PK data for Colombian adult patients (Table II) were used to develop and refine the miltefosine PBPK model for adults, detailed in the flowchart in Fig. 2.

**Table II:** Development Of Miltefosine PBPK Model for Adults Based On the Clinical Trial in Colombia

		Clinical trial	Simulation					
Total no. of patients		22	22					
Demographic data	Female patients, n(%)	12(55)	12(55)					
	Ethnicity, n	Colombian & Mestizo	North American African-American					
	Age (years)	34 (21–51)	34 (21–47)					
	Body weight (Kg), mean (range)	70.8(50.4–102)	69 (51–103)					
	Height (cm)	165 (152–182)	165.6 (145–194)					
Daily dose of miltefosine (mg/kg/day)		2.5	2.5					
PBPK Model parameters			Initial values in Table I		Optimal values after ASA tests in Table I			
			Prediction	FE	Prediction		FE	
Plasma PK data	C_max_ (µg/mL), mean (range)	31.9 (17.2–42.4)	25.6 (10.0–47.0)	0.8	39.4 (11.3–98.2)		1.2	
	T_max_ (days), mean (range)	16 (13.8–28.1)	28	1.7	28		1.7	
	t_½_ (days), mean (range)	34.4 (9.5–46.2)^[a]^	12.0 (10.6–14.8)	N/A	4.87 (4.3–6.1)		N/A	
	AUC_d0-28_ (µg⋅day/mL), mean (range)	628 (213–861)	491 (244–799)	0.7	886 (307–1853)		1.4	
	AUC_d0-∞_ (µg⋅day/mL), mean (range)	880 (427–1206)	943 (284–2766)	1.0	1159 (319–3496)		1.3	
PD model parameters					Initial value α		Optimal value α	
					Prediction	FE	Prediction	FE
Intracellular PK data	C_max_ (µg/mL), mean (range)	71.5 (40.0–150)			96.7 (27.9- 240.9)	1.3	77.4 (22.3–192.7)	1.0
	T_max,_ (days), mean (range)	27.5 (13.8–30.0)			28	1.0	28	1.0
	AUC_d0-28_ (µg⋅day/mL), mean (range)	1316 (625–2667)			2174 (754–4547)	1.6	1739 (604–3637)	1.3

[a] terminal half-life, *N/A* no applicable

To better match the *in silico* clinical trials to the relevant actual clinical trial [10], virtual trial subjects were generated using the Sim-North American Hispanic_Latino population. The number of subjects employed in the* in silico* clinical trial was 22, aged between 21 to 51 years, with 12 females. The Simulator default equations for height and weight were adjusted manually to achieve the required mean values of height and weight in the clinical study. In the meantime, the coefficient of variation (CV) of height or weight was also adjusted to get the best match for the range. Details can be found in Table S2. Comparison of the demographic data (i.e., age, weight, height, and proportion of females) of the *in silico* virtual and clinical populations is shown in Table II, indicating that they matched very well. In the trial design, a dose of 2.5 mg/kg/day was administered to the virtual populations for 28 days. The oral route, with a 250 mL drink of water with dose, was utilized and in the fed state to reduce miltefosine GIT side effects [12]. Samples were collected from the virtual participants at a uniform interval of 24 h daily for 210 days as the clinical literature reports that miltefosine persists in the system for 5 to 6 months due to its long half-life [1]. Ten virtual repeat trials of the study design were selected to better ensure the participants used in the actual clinical trials were represented in the simulation and to consider study power.

**Fig. 3:**
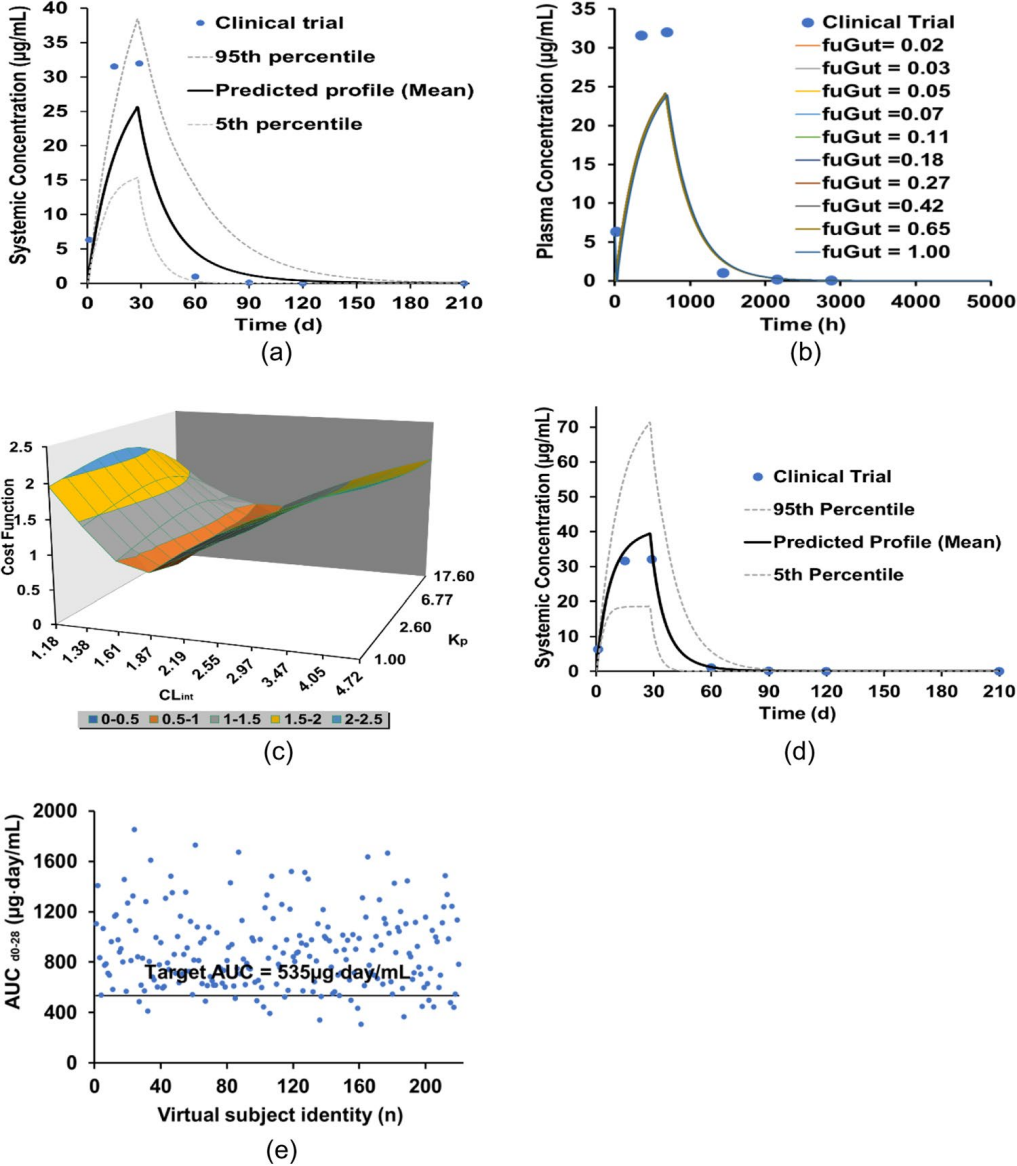
Development of miltefosine PBPK model for adults; (**a**) miltefosine concentration–time profile based on the initial parameters shown in Table I; (**b**) ASA for f_uGut_ at different values; (**c**) miltefosine PK value cost function as a function of combined K_p_ Scalar and CL_int_; (**d**) miltefosine concentration–time profile based on the optimal parameters; (**e**) AUC_d0-28_ distribution of the virtual subjects based on the optimal parameters. Note: The clinical trial data were extracted directly from the reference.

Based on the initial (unoptimized) model parameters (Table I), the predicted miltefosine mean concentration–time profile with the upper (95%) and lower (5%) percentiles are shown in Fig. 3, indicating that miltefosine continues to accumulate until the end of treatment at 28 days due to slow plasma clearance (mean complete elimination in more than 120 days). The predicted PK parameters and their ranges are shown in Table II. Compared to clinical trial data, the predicted PK parameters (C_max_, T_max_, and AUC) were within the defined acceptance criteria of 0.5 to 2 FE. It is worth noting that t_½_ was not considered here because the values recorded were based on the terminal elimination half-life. However, “steady state” was not reached by the end of the 28-day treatment in the simulations, which is significantly different to the clinical trial results [Fig. 3(a)]. It was expected that miltefosine plasma concentration would increase for the first two weeks of treatment and then reach ‘‘steady state’’ maintained until the end of treatment at 28 days [48]. Hence, the model parameters were refined (Table I).

In the Methods, three parameters, i.e., f_uGut_, K_p_ Scalar and CL_int_, were identified as candidates for optimization. Firstly, ASA was performed for f_uGut_, assessing a range from the default value of 1 to 0.02 (plasma f_u_). A previous study has suggested that f_uGut_ can be set as the free fraction of the drug in plasma or blood depending on various assumptions [35]. The simulations show that a change in f_uGut_ has little effect on the predicted concentration–time profile profiles [Fig. 3(b) and detailed PK values in Table S3 in the supporting materials]. Thus, f_uGut_ was kept as the default value of 1 in the miltefosine PBPK model.

ASA was then conducted to investigate the combined effects of changes to both the K_p_ Scalar and CL_int_ on the predicted concentration–time profile of miltefosine. The range of K_p_ Scalar values was from the default value of 1 (no effect on Kp prediction) to the initial adjusted value of 17.6 whilst the range of CL_int_ values was from 1.18 (i.e., half time of the initial value of 2.36) to 4.72 (i.e., 2 times the initial value of 2.36). For ASA, ten log-distributed steps were selected for both parameters. Thus 100 combinations of K_p_ Scalar and CL_int_ were tested. In order to determine optimal combinations, the following cost function was used to determine the best fit between the predicted and clinic trial PK values as

6 $$\underset{{\mathrm{K}}_{\mathrm{p}},{\mathrm{CL}}_{\mathrm{int}}}{\mathrm{min}}\mathrm{E}\left({\mathrm{K}}_{\mathrm{p}},{\mathrm{CL}}_{\mathrm{int}}\right)=\mathrm{abs}\left(\frac{{\mathrm{C}}_{\mathrm{max}}-{\widehat{\mathrm{C}}}_{\mathrm{max}}}{{\mathrm{C}}_{\mathrm{max}}}\right)+\mathrm{abs}\left(\frac{{\mathrm{T}}_{\mathrm{max}}-{\widehat{\mathrm{T}}}_{\mathrm{max}}}{{\mathrm{T}}_{\mathrm{max}}}\right)+\mathrm{abs}\left(\frac{{\mathrm{AUC}}_{\mathrm{d}0-\infty }-{\widehat{\mathrm{AUC}}}_{\mathrm{d}0-\infty }}{{\mathrm{AUC}}_{\mathrm{d}0-\infty }}\right)$$

where ${\widehat{\mathrm{C}}}_{\mathrm{max}}$, ${\widehat{\mathrm{T}}}_{\mathrm{max}}$ and ${\widehat{\mathrm{AUC}}}_{\mathrm{d}0-\infty }$ are the predicted PK values in plasma.

The cost function (Eq. (6)) for the different combinations of K_p_ Scalar and CL_int_ values is shown in Fig. 3(c), indicating that the cost function range was from 2.145 to 0.765 (detailed in Table S4 in the supporting materials). Ten of the lowest combinations of K_p_ Scalar and CL_int_ in the ASA test (Table S5 and Figure S1 in the supporting materials) were selected to run the PBPK model to obtain the optimal combination of K_p_ Scalar = 4.92 and CL_int_ = 1.87, based on the FE values in Eq. (4) and CR in Eq. (5). The predicted mean value of the miltefosine plasma concentration − time profile based on the optimal parameters is shown in Fig. 3(d), indicating that the miltefosine exposure in plasma for Colombian adult patients can be predicted accurately, where the miltefosine plasma concentration reached a ‘‘steady state’’ at day 14 and was increased slowly until the end of treatment at 28 days. The predicted PK parameters (C_max_, T_max_, and AUC) were within the acceptance criteria of 1.2 to 1.7-fold error (Table II). In particular, the CR of 91.8% has been predicted based on the plasma exposure of AUC_0-28_ [Fig. 3(e)], which agrees with the observed CR of 100% in a clinical trial.

**Fig. 4:**
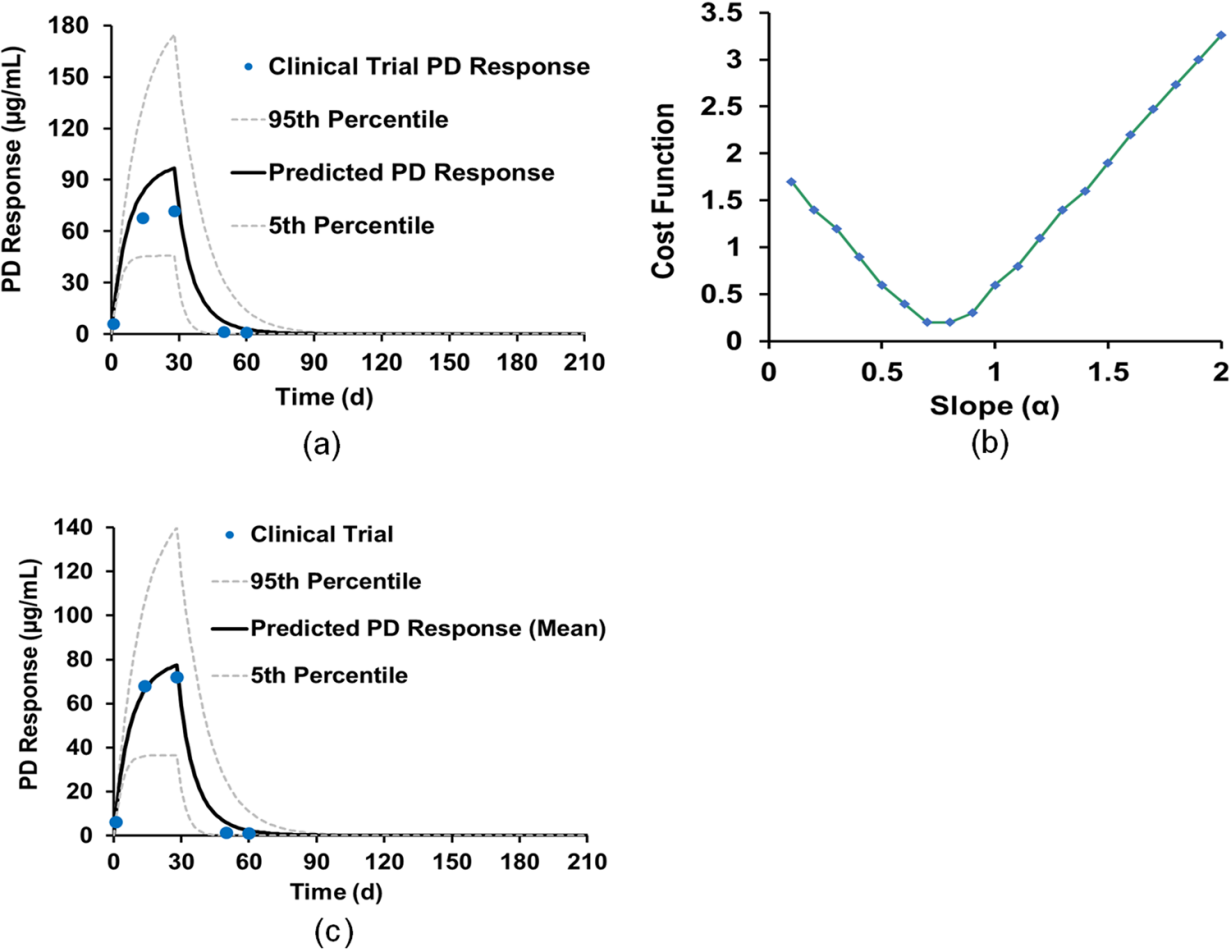
Development of miltefosine PD model; (**a**) miltefosine concentration–time profile in the intracellular PBMCs based on the PBPK parameters shown (Table I); (**b**) miltefosine PD value cost function as a function of the activation constant α; (**c**) miltefosine concentration–time profile in the intracellular PBMCs based on the optimal activation constant α.

Finally, the miltefosine concentration profile in PBMCs was predicted using the Simcyp Simulator PD model (Materials and Methods). Based on Eq. (3), the value of ${C}_{PL}$ was predicted using the optimal PBPK model developed above. Based on the initial activation constant α of 1 in Table I, the predicted miltefosine mean concentration–time profile in the intracellular PBMCs along with the upper (95%) and lower (5%) percentiles are in Fig. 4(a), showing that the predicted miltefosine concentration profile in PBMCs was higher than that of the clinical trial. A direct comparison of the predicted and clinical trial PK parameters is shown in Table II. Thus, an ASA was conducted to optimize the activation constant α with the range of 2 to 0.1 at a uniform step-size interval of 0.1. The cost function defined by Eq. (5) was used in the test. The cost function as a function of the activation constant α is shown in Fig. 4(b), in which minimal cost function was achieved where α is 0.7 and 0.8 (Table S6 and Figure S2). Further simulations were conducted showing that α = 0.8 was the optimal value. The comparison of the predicted and experimental miltefosine intracellular concentration − time profiles with final optimized α of 0.8 is shown in Fig. 4(c) and the detailed PK value comparison is shown in Table II, indicating that the C_max_, AUC_d0-28_, and T_max_ of miltefosine in PBMCs can be predicted accurately.

#### Assessment of the Model Predictability for Different Ethnic Adult Populations

In order to assess the predictability of the developed PBPK model for different ethnic adult populations, simulations based on the optimised input parameters (Table I) for the clinical trials of miltefosine involving Dutch military personnel [32], Nepalese adults [13] and East African adults [9, 16] were conducted. The virtual subjects were generated from the built-in databases of Sim-NEurCaucasian, Sim-North American Asian and Sim-North American African American of the Simcyp human simulator. The virtual subjects’ demographics (detailed height and weight functions given in Tables S7 in the supporting materials) were selected based on the actual clinical trial data. It is worth noting that there are significant differences in the demographic data (Table III) of the virtual subjects with the actual clinical trial patients in the Nepalese and East Africa simulations because the clinical trial subjects includes both adults and children. Ten multiple trials of each of the study designs were selected. The fixed doses of 150 and 100 mg/day were administered orally in the Dutch military personnel and Nepalese adult simulations, respectively, whilst a dose of 2.5 mg/kg/day was administered orally in the African adult simulation, all of whom were treated for 28 days.

**Table III:** Miltefosine PBPK Model Predictions for Different Ethnic Adult Populations

		Europe			Nepal			East Africa		
		Clinical trial	Simulation		Clinical trial*	Simulation		Clinical trial	Simulation	
Total no. of patients		31	31 × 10 Trials		81	50 × 10 Trials		29	30 × 10 Trials	
Demographic data	Female patients percentage n(%)	3.2	3.2		38.2	38.2		15	50	
	Ethnicity, n	Dutch military personnel	Sim-NEurCaucasians		Nepalese	Sim-North American Asian		Kenya and Sudan	Sim-North American Hispanic_Latino	
	Age (years)	24 (23–29)	25.9 (23–29)		20 (2–65)	41.3 (18–65)		13 (7–41)	29.4 (18–41)	
	Body weight (Kg), mean (range)	85 (78–89)	85.2 (69–89)		40 (8–56)	50.7 (43–57)		33.5 (16–65)	60.5 (52–72)	
	Height (cm)	184 (180–188)	184.0 (168–187)		147 (75–172)	165.0 (151–176)		150 (107–185)	165.6 (152–180)	
dose		150 (mg/day)			100 (mg/day)			2.5 (mg/kg/day)		
Patients with treatment failure percentage n (%)		NR	29		40	33		23	13	
			Prediction	FE		Prediction	FE		Prediction	FE
Plasma PK data	C_max,_ (µg/mL), mean (range)	30.8 (0.007—51.6)	30.2 (6.3–77.5)	0.9	35.3 (11.6–120)	28.8 (6.2–90.1)	0.8	NR	35.4 (7–94)	
	T_max,_ (days), mean (range)	28	28	1	NR	28		NR	28	
	t_½_ (days), mean (range)	7.05 (5.45 -9.10)	5.22 (4.4–5.6)	0.7	6.26 (4.18–9.27)	4.96 (4.54–5.44)	0.8	7.18 (5.35–10.9)	4.35 (4.05–4,882)	0.6
	AUC_d0-28_ (µg⋅day/mL), mean (range)	NR	672.5 (171.2–1421.8)		724 (265–2260)	648.6 (167.6–1660.2)	0.8	497 (191–767)	815.6 (190.5–1805.5)	1.6
	AUC_d0-∞_ (µg⋅day/mL), mean (range)	NR	895.4 (176.5–3112.4)		1140 (340–4200)	848.0 (176.2–3623.2)	0.7	812 (237–1482)	1024.9 (196–3216)	1.2

*NR* no report

*: The observed data are not true observed or raw data as they are not available. Thus these data were obtained based on estimation with the corresponding popPK mode (Table III in [15])

**Fig. 5:**
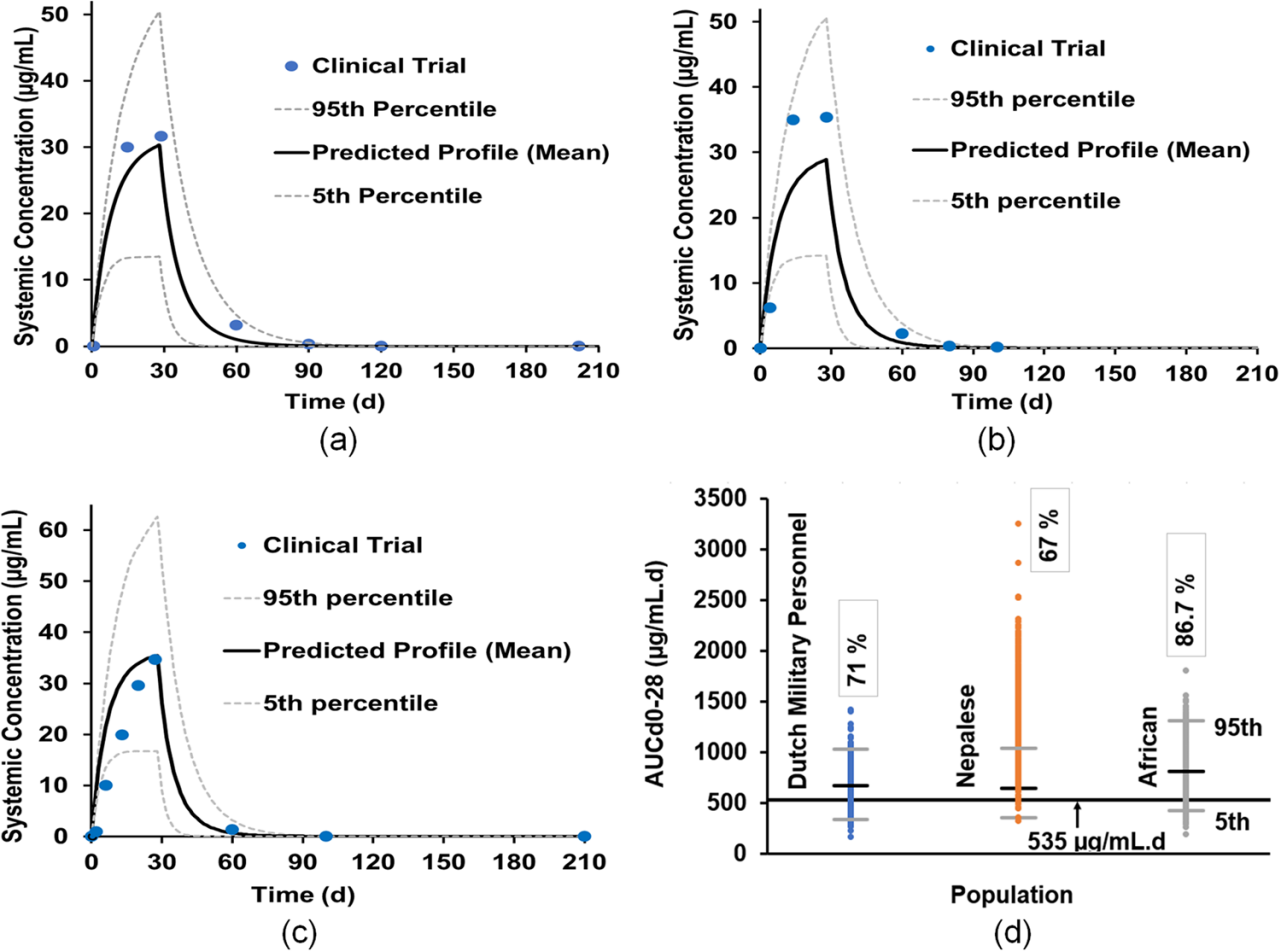
Validation results of miltefosine PBPK model for adult validation results: (**a**) simulated and observed (solid circles) mean plasma concentration–time profiles of Dutch military personnel; (**b**) simulated and observed (solid circles) mean plasma concentration–time profiles of Nepalese adults; (**c**) simulated and observed (solid circles) mean plasma concentration–time profiles of Africa adults; (**d**) AUC_d0-28_ distribution of the virtual subjects.

The simulated mean plasma concentration–time profiles of miltefosine in European, Nepalese and Africa adults were in good agreement with the clinical trial results, where all the observed data points were within the 5th and 95th percentiles of the simulated data in Fig. 5(a)-(c) and the observed PK parameters (including the elimination half-life T_1/2_) were within the acceptance criteria of 0.5 to twofold error in Table III.

Additionally, the simulations also provided the PK results for each of the individual simulated virtual subjects. The AUC_d0-28_ distributions in the three simulations are shown in Fig. 5(d). 71% of the virtual NEurCaucasians were observed to reach the target PK value of 535 µg⋅day/mL. A cure rate was not provided in the clinical trial [32]. However, based on a separate clinical trial in Iran, the cure rate was 81.3% [49]. For the North American Asian virtual trial, about 33% of virtual subjects were observed to have AUC_d0–28_ less than 535 μg.day/mL, which was similar to the report with a treatment failure of 40% [15], whilst about 13.3% of the virtual North American African-Americans having AUC_d0–28_ less than 535 μg.day/mL were observed in comparison with 23.0% in the clinical trial [16].

Furthermore, miltefosine concentration–time profiles in PBMCs (Figure S3) were also available in the simulations, but, they cannot be validated due to lack of data in the clinical trials. The predictions of the bioavailability and dose fraction absorbed for various populations are given in Table S11 in the supporting materials.

### Miltefosine PBPK Model for Children

**Fig. 6:**
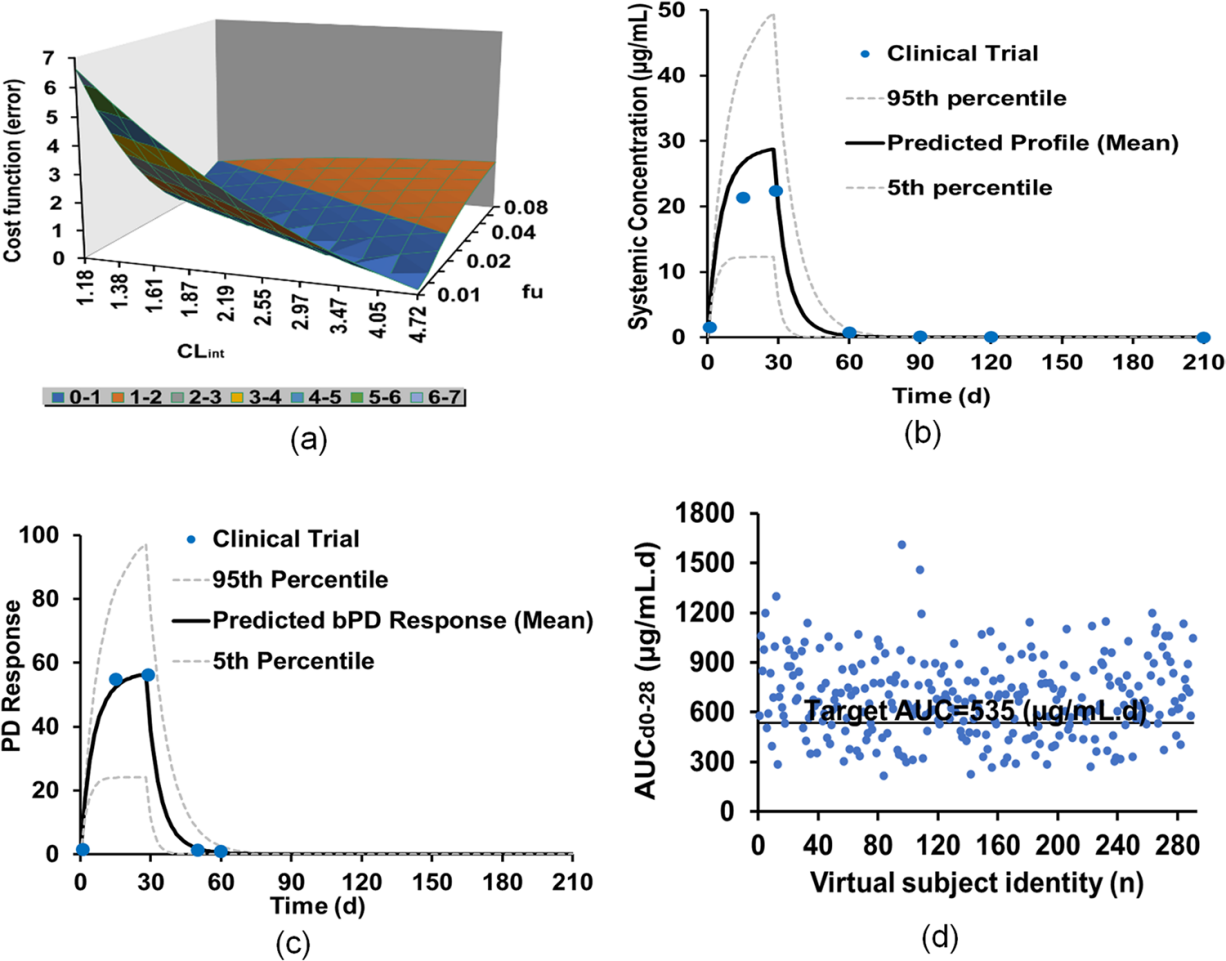
Development of miltefosine PBPK model for children: (**a**) miltefosine PK value cost function as a function of a combination of F_up_ and CL_int_; (**b**) miltefosine concentration–time profile in plasma of paediatric Colombian based on the optimal parameters; (**c**) miltefosine concentration–time profile in PBMCs of paediatric Colombian based on the optimal parameters; (**d**) AUC_d0-28_ distribution of the virtual subjects based on the optimal parameters.

Following finalization of the miltefosine PBPK model for adults, the system-specific inputs are modified to develop the paediatric PBPK model, while drug-specific inputs remain unaltered. In the model, the virtual paediatric subjects were generated from the database of Sim-Paediatric within Simcyp. The similar demographics of the virtual subjects (Table IV) as those of the actual clinical trial data were obtained by optimizing the height and weight functions (Table S8). As described in Section of Population and Trial Design, the fraction of the unbound drug in plasma f_u_ and clearance CL_int_ were optimised based on the clinical PK data of Colombian children with cutaneous leishmaniasis [10, 31]. The range of f_u_ values was from 0.01 to 0.08 whilst the range of CL_int_ values was from 1.18 to 4.72, using 10 steps for each of the parameters with log-distributed step-size. According to the cost function surface [Fig. 6(a)] calculated in Eq. (6), the optimal combination is f_u_ = 0.04 and CL_int_ = 1.18. The predicted mean miltefosine concentration − time profile based on the optimal parameters is shown in Fig. 6(b), indicating that the miltefosine exposure in plasma for Colombian paediatric patients can be predicted accurately. The FE values in Table IV were within the range of 0.5 to 2.0, indicating that the simulations of the clinical trials were successful. The miltefosine concentration profile in PBMCs for the paediatric simulation was predicted based on the adult activation constant α = 0.8 without modification [Fig. 6(c)]. The detailed PK value comparison of Colombian paediatrics is shown in Table IV, indicating that the C_max_, AUC_d0-28_, and T_max_ of miltefosine can be predicted accurately.

**Table IV:** Comparison of Miltefosine Paediatric PBPK Model Predictions With the Clinic Trial Data

		Colombian paediatric			East Africa		
		Clinical trial	Simulation		Clinical trial	Simulation	
Total no. of patients		29	29 × 10 trials		21	21 × 10 trials	
Demographic data	Female patients percentage n(%)	41.4	41.4		24	24	
	Ethnicity, n	Afro-Colombian & Mestizo	Sim-Paediatric		Kenya, 7 (33); Sudan, 14 (67)	Sim-Paediatric	
	Age (years)	8(2–12)	7 (2–12)		10(7–12)	9.5 (7–12)	
	Body weight (Kg), mean (range)	26.5 (12.6–45.9)	26.4 (9.2–63)		24 (16–65)	25 (10.3–54.1)	
	Height (cm)	126 (92–153)	126 (92–158)		135 (107–153)	135.6 (121.5–153.6)	
dose		2.5(mg/kg/day)			2.5 (mg/kg/day)		
Patients with treatment failure percentage n (%)		17.2	26.5		41	43.8	
			Prediction	FE		Prediction	FE
Plasma PK data	C_max,_ (µg/mL), mean (range)	22.7 (17.0–29.3)	28.7 (8.1–81.2)	1.2	19.9 (14.4–37.7)	24.4 (6.4–74)	1.2
	T_max,_ (days), mean (range)	27.8 (13.9–28)	28	1.0	NR	28	-
	t_½_ (days), mean (range)	NR	3.7 (3.3–4.0)	-	7.02 (4.02–8.45)	3.21 (1.6–3.9)	0.5
	AUC_d0-28_ (µg⋅day/mL), mean (range)	448 (304–583)	685.4 (219.2–1609.6)	1.5	321.9 (261.2 – 478.0)	590.5 (173.1–1484.3)	1.8
	AUC_d0-∞_ (µg⋅day/mL), mean (range)	652 (438–832)	818.2 (228.5–2623.6)	1.2	550.5 (404.1- 891.6)	693.6	1.2
Intracellular PK data	C_max,_ (µg/mL), mean (range)	55.6 (19.8–382)	56.4 (16–159.3)	1.0	NM	48 (12.5–145.2)	-
	T_max,_ (days), mean (range)	23.2 (13.0–28.0)	28	1.2	NM	28	-
	AUC_d0-28_ (µg⋅day/mL), mean (range)	964 (393–4552)	1345.4 (430.2–3159.4)	1.3	NM	1159.1 (339.7–2913.5)	-

**Fig. 7:**
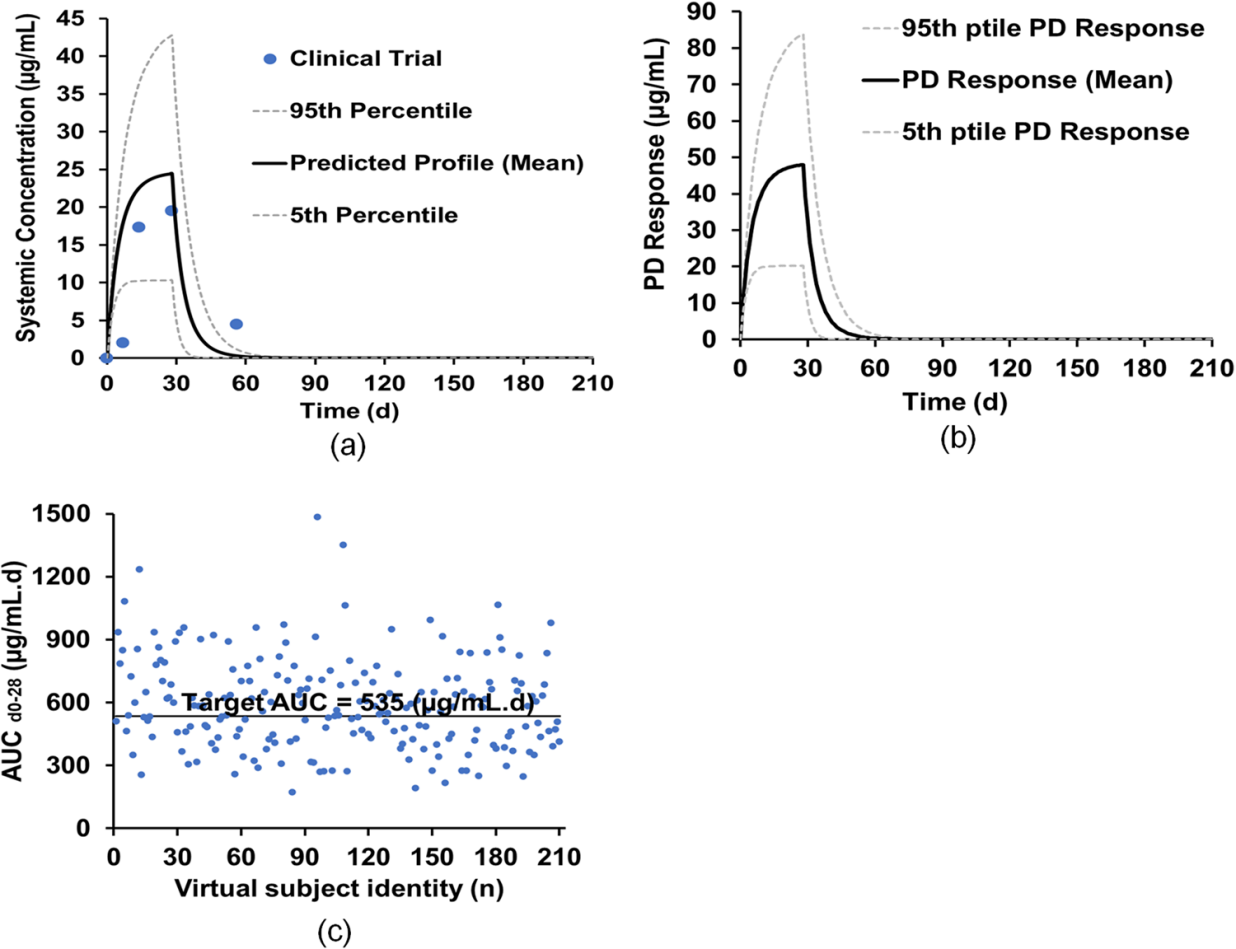
Validation of miltefosine PBPK model for children: (**a**) miltefosine concentration–time profile in plasma of East African paediatrics; (**b**) miltefosine concentration–time profile in PBMCs of East African paediatrics; (**c**) AUC_d0-28_ distribution of the virtual subjects.

The distributions of the plasma exposures of AUC_d0-28_ of the virtual subjects are shown in Fig. 6(d). The CR in Colombian Children is 73.4%, closely matching the findings from a clinical trial in Colombia for paediatrics at 82.8% (Table IV). Furthermore, the developed paediatric PBPK model of miltefosine has been used to predict the PK results for a clinical trial with East African children [17]. The closest match to the East African paediatric subjects was North-European Paediatric Population within Simcyp. The height equation was left at the default setting as it predicted the height of the virtual paediatrics, while the CV was adjusted manually to get the required distribution (Table S8). Additionally, the equation and CV for body weight was adjusted to predict the weight of the virtual East African paediatric subjects (Table S8). Figure 7(a)&(b) show the predicted miltefosine plasma and intracellular concentration–time profiles in the virtual North-European Paediatric Populations following multiple doses of 2.5 mg/kg/day for 28 days, indicating a close match in the PK parameters for the clinical trial and prediction. The FE values (Table IV) were within the range of 0.5 to 2.0, indicating that the simulations of the clinical trials were successful. The distributions of the plasma exposures of AUC_d0-28_ of the virtual subjects are shown in Fig. 7(c), showing that the CR of Eastern African Children is 56.1% which closely matches the observed CR of 59%.

### Dose-Exposure–Response Relationships in Different Populations

**Fig. 8:**
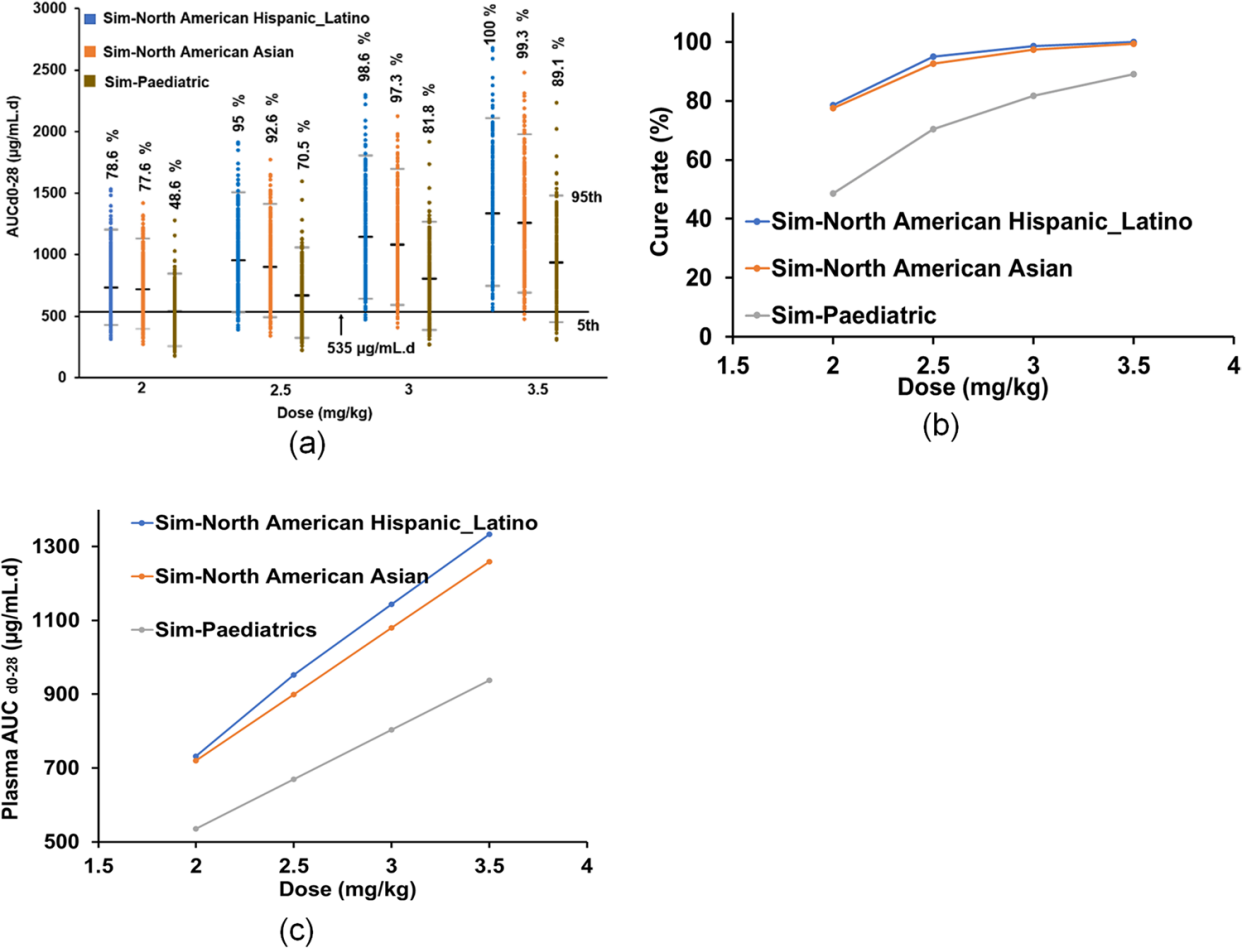
Dose-exposure–response relationships in different populations: (**a**) AUC_d0-28_ distributions of populations under different doses; (**b**) CR comparison (**c**) AUC_d0-28_ vs dose curve.

Based on the developed adult and paediatric miltefosine PBPK models, the dose-exposure–response relationships of miltefosine for adult and paediatric populations have been investigated. Various doses of miltefosine at 2 mg/kg, 2.5 mg/kg, 3 mg/kg and 3.5 mg/kg were administered to virtual participants, including two adult populations with the age range of 18 to 65 years generated by Sim-North American Hispanic_Latino and Sim-North American Asian and one paediatric population with the age range of 2 to 12 years generated by Sim-Paediatric. Thirty subjects, 50% female and 10 multiple trials of each study design were selected. Details of the demographics for each population and the exposure-time curves for corresponding virtual subjects under different doses are shown in Table S9 and Figure S4. There is no significant difference in the distribution of the miltefosine exposure of AUC_d0-28_ in Fig. 8(a) between the Sim-North American Hispanic_Latino and Sim-North American Asian adults at different doses, showing comparable CRs of the two ethnic adult populations in Fig. 8(b). Although a linear relationship between the dose and exposure was observed in Fig. 8(c), the dose–response relationship was nonlinear in Fig. 8(b). The CR at 2 mg/kg dose is 79% (Sim-North American Hispanic_Latino) or 79.5% (Sim-North American Asian), which is low. It can be increased above 95% for both adult populations if the dose is increased to 2.5 mg/kg from 2 mg/kg. There is no significant increase in the CR with further increasing the dose, showing that 2.5 mg/kg is the optimal dose for the adult treatment.

In contrast, a lower miltefosine exposure in paediatrics was observed in Fig. 8(a) compared with the adults. The CR of paediatrics was just 48.6% at a dose of 2 mg/kg, and it was increased to 70.4% or 81.8% when the dose was increased to 2.5 mg/kg or 3 mg/kg in Fig. 8(b). Even with a dose of 3.5 mg/kg, the overall CR of paediatrics was 89.1%.

## Discussion

As the only oral drug currently used to treat leishmaniasis, miltefosine has an important role in eliminating leishmaniasis as a public health problem that affects millions of the poorest populations in the world. However, clinical outcomes of miltefosine treatment vary significantly, depending not only on the dose regimen selection but also on the nature of the treated population, notably age. Thus, it is essential to establish a population-based dose-exposure–response relationship for various regimens of miltefosine to determine an optimal dosing regimen to guide the treatment of leishmaniasis. In this work, the cure rate analysis as the response model of miltefosine was conducted based on the proposed PK target for cutaneous leishmaniasis of AUC_d0-28_ > 535 µg⋅day/mL [31]. In visceral leishmaniasis, the time that the plasma concentration was above the *in vitro* susceptibility EC_90_ (t > EC_90_ for Eastern Africa) or above 10xEC_50_ (for Nepal) was proposed for the probability of cure and relapse hazard [12]. For simplicity, the cure rate of a simulation was based on the percentage of the number of individual virtual subjects with AUC_d0-28_ > 535 µg⋅day/mL in the virtual subjects in Eq. (5) for both cutaneous leishmaniasis and visceral leishmaniasis patients.

The development of the PBPK model of miltefosine provides the opportunity not only to predict the PK parameters and concentration–time profiles in plasma and PBMCs but also to gain mechanistic insight into the compound’s absorption and elimination. The exposure of miltefosine is determined by the activity of phospholipase D because the enzyme is the only elimination pathway for the drug. Previous studies have shown that phospholipase D activity is closely related to the development of obesity and ageing [50, 51]. Therefore, the changes in the expression of phospholipase D enzymes with the population weight and age are needed in the model. As the Simcyp PBPK Simulator used in the work has incorporated covariates and interindividual variability in systems parameters (e.g., body weight, age, blood flow rate, and metabolism rate), the weight factor is inherently considered in the developed PBPK model. However, the changes in the expression of phospholipase D enzymes with age is not available in the software. Therefore, it is necessary to assemble both adult and paediatric PBPK models separately.

The adult PBPK model of miltefosine was developed and refined using clinical pharmacokinetic data of miltefosine in the adult cutaneous leishmaniasis patients in Colombia [10], where the characteristics of the virtual subjects were matched closely with those in the clinical trial; the model was able to accurately simulate miltefosine exposure distributions in plasma and PBMCs within the defined acceptance criteria of 0.5 and 2 FE. To assess performance in different ethnic adult populations, the developed PBPK model was used to predict the PK values of European [10], Nepalese [15] and African [9, 16] adult patients, in which suitable virtual populations were generated from the databases within Simcyp. It is shown that both the PK data and CRs of the European, Nepalese, and African adult patients in the clinical trials can be predicted accurately, demonstrating that ethnic difference is not a key consideration for miltefosine treatment. A comparative result of the predicted dose-exposure–response of the different simulated ethnicities under different dose regimes of miltefosine treatment (Fig. 8) further supports the dose-selection strategy is the main factor to determine the clinical outcome of miltefosine treatment. Although fixed dosing is potentially more convenient than weight-based dosing for both patients and physicians, a lower CR of the miltefosine treatment in the clinical trial for either European or Nepalese adult patients at fixed dosing (i.e., 150 mg/day or 100 mg/day) was observed due to inter-subject variability, resulting in under exposure to miltefosine. At an optimal weight-based dosing strategy of 2.5 mg/kg, the CR of the miltefosine treatment can be improved to 92.6% and above in adults (Fig. 8).

The paediatric PBPK model of miltefosine was obtained by optimising the age-dependent parameters of drug protein binding and intrinsic clearance from the adult model. Whilst there was no difference in exposure levels in virtual adult populations for a given dose, a significantly lower miltefosine exposure was observed in virtual paediatrics, resulting in a lower CR (Fig. 8). These simulation results were consistent with those observed in the clinical trials. The developed PBPK models suggest that the difference in miltefosine exposure between children and adults is associated with plasma protein distribution determined by the unbound fraction of miltefosine in plasma. Generally, the plasma protein binding of a drug gradually increases with age because Human Serum Albumin (HSA) concentrations are close to adult levels at birth (75 -80%), whereas alpha 1-acid glycoprotein (AAG) concentrations are initially half that of adults [52]. As miltefosine is highly bound to plasma proteins such as HSA and low-density lipoprotein in the range of 96 to 98% in adults [12, 31], it was expected that children have higher f_u_, the fraction unbound miltefosine in plasma. Indeed, the optimal value of f_u_ in paediatric PBPK model was 0.04 which is twice that for adults. Consequently, this resulted in different exposure patterns affecting the distribution and clearance between children and adults.

It has to be stressed that a higher weight-based dosing strategy can improve the CR in paediatrics. For example, at a weight-based dose of 3.5 mg/kg, the CR was 89.1% in the virtual paediatric population (Fig. 8). However, it is still lower than the dosing strategy based on FFM, which achieved a 95% CR of the treatment in the clinical trial [17]. A further simulation using the virtual Sim-Paediatric population with a higher dose of 3.9 mg/kg showed a CR of 91.8% was observed (Figure S5 and Table S10). This suggests that there was no significant difference when 3.5 and 3.9 mg/kg of miltefosine was administered to paediatric subjects.

## Conclusion

In this work, mechanistic population-based PBPK models have been developed to study the dose-exposure–response relationship of miltefosine in* in silico *clinical trials and to evaluate differences of the treatment in different population groups, particularly children and adults. The Simcyp Population pharmacokinetics platform was employed to predict miltefosine exposure in plasma and PBMCs in virtual populations under different dosing regimens. The cure rate of a simulation was based on the percentage of the number of individual virtual subjects with simulated AUCd_0-28_ > 535 µg⋅day/mL. It was shown that both adult and paediatric PBPK models of miltefosine can be developed to predict the PK reported from clinical trials accurately. There is no significant difference in the predicted dose-exposure–response of the miltefosine treatment for different simulated ethnicities under the same dose regime. The clinical outcome of the miltefosine treatment is mainly determined by the dose-selection strategies. A lower CR of the miltefosine treatment was predicted in paediatrics due to the lower miltefosine exposure observed in virtual paediatrics compared to adult virtual populations receiving the same doses. The simulation results were consistent with those of the clinical trials. The mechanistic PBPK model suggested that the fraction of unbound miltefosine in plasma was responsible for a higher probability of failure in paediatric subjects because of the difference in the distribution of plasma proteins between adults and paediatrics.

In summary, the miltefosine dosing strategy plays a key role in successfully treating leishmaniasis. It is expected that the developed model and approach could be used to determine an optimal miltefosine dose regime in future clinical trials.

### Supplementary Information

Below is the link to the electronic supplementary material.

Supplementary file1 (DOCX 1068 KB)

## Data Availability

The datasets used are available from the corresponding author upon reasonable request.
