# Supplementary material for: Assessing Dose-Exposure–Response Relationships of Miltefosine in Adults and Children using Physiologically-Based Pharmacokinetic Modeling Approach
Source: Pharm Res. 2023 Oct 10;40(12):2983–3000. doi: 10.1007/s11095-023-03610-0 (PMC10746618; doi:10.1007/s11095-023-03610-0)
Supplement: Supplementary file 1 — Supplementary file1 (DOCX 1068 KB) [file 11095_2023_3610_MOESM1_ESM.docx]

**Supporting materials**

**Assessing Dose-Exposure-Response Relationships of Miltefosine in Adults and Children using Physiologically-Based Pharmacokinetic Modeling Approach**

Shadrack J. Madu^1^, Ke Wang^1^, Siri Kalyan Chirumamilla^2^, David B. Turner^2^, Patrick G. Steel^3^, and Mingzhong Li^1^^[[1]](#footnote-1)^*

*^1^School of Pharmacy, De Montfort University, Leicester, LE1 9BH, U.K.*

*^2^Certara UK Limited, Simcyp Division, Sheffield S1 2BJ, U.K.*

*^3^Department of Chemistry, Durham University, Durham, DH1 3LE, U.K.*

**Table S1:** Summary of the PK data obtained from different clinical trials

|  | | **Colombia** | | **European** | **Nepalese** | **East Africa (Kenya & Sudan)** | | **Children in East Africa (Kenya, Sudan and Uganda)** | |
| --- | --- | --- | --- | --- | --- | --- | --- | --- | --- |
|  | | **Adults** | **Children** | **Adults** | **Adults** | **Adults** | **Children** | **Children** | **Children** |
| Total no. of patients | | 22 | 29 | 31 | 81 | 29 | 19 | 21 | 30 |
| Demographic data | Female patients, n(%) | 12(54.5) | 21(41.4) | 1 (3.2) | 31 (38.2) | 7 (14.6) | | 5 (24) | 8 (27) |
|  | Ethnicity, n | Afro-Colombian 17 & Mestizo 5 | Afro-Colombian 21 & Mestizo 8 | Dutch military personnel | Nepalese | Kimalel in Kenya, 24; Kassab in Sudan, 7 and Dook in Sudan, 17 | | Kenya, 7; Sudan, 14 (67) | Kenya, 21; Uganda, 9 |
|  | Age (years) mean (range) | 34 (21-51) | 8(2-12) | 24 (23–29) | 20 (2–65) | 13 (7–41) | | 10(7–12) | 7 (4-12) |
|  | Body weight (Kg) mean (range) | 71.1(50.4-102) | 26.5 (12.6-45.9) | 85 (78–89) | 40 (8–56) | 33.5 (16–65) | | 24 (16–34) | 21.8 (13.0-29.5) |
|  | Height (cm) mean (range) | 165 (152-182) | 126 (92-153) | 184 (180–188) | 147 (75–172) | 150 (107–185) | | 135 (107–153) | 125 (99–145) |
| dose | | 2.5 (mg/kg/day) | 2.5 (mg/kg/day) | 150 (mg/day) | 100 (mg/day) | 2.5 (mg/kg/day) | | 2.5 (mg/kg/day) | 3.2 median (mg/kg/day) |
| Patients with treatment failure, n (%) | | 0(0) | 5(17.2) | NR | 16 (21%) | 13 (28) | | 9 (41) | 3(10) |
| Plasma PK data | C_max_ (µg/mL), mean (range) | 31.9 (17.2-42.4) | 22.7 (17.0-29.3) | 30.8 (0.007 - 51.6) | 35.3 (11.6–120) | NR | NR | 19.9 (14.4-37.7) | 21.0 (15.5-28.6) |
|  | T_max_ (days), mean (range) | 16.0 (13.8-28.1) | 27.8 (13.9-28) | 28 | NR | NR | NR | NR | NR |
|  | t_½_ (days), mean (range) | 34.4 (9.5-46.2) ^[a]^ | 37.1 (7.4-47.0) ^[a]^ | 7.05 (5.45 -9.10) ^[b]^ | 6.26 (4.18–9.27) ^[b]^ | 7.18 (5.35–10.9) ^[b]^ | 7.02 (4.02–8.45) ^[b]^ | 7.02 (4.02–8.45) ^[b]^ | NR |
|  | AUC_d0-7_ (µg⋅day/mL), mean (range) | NR | NR | NR |  |  |  | 13 (3.07-42.87) | 22.85 (4.14-96.02) |
|  | AUC_d0-28_ (µg⋅day/mL), mean (range) | 628 (213-861) | 448 (304-583) | NR | 724 (265–2260) | 497 (191–767) | 352 (232–593) | 321.9 (261.2 – 478.0) | 385.5 (271.0-651.7) |
|  | AUC_d0-∞,_ (µg⋅day/mL), mean (range) | 880 (427-1206) | 652 (438-832) | NR | 1140 (340–4200) | 812 (237–1482) | 545 (314–1080) | 550.5 (404.1- 891.6) | 588.6 (396.0-875.7) |
| Intracellular PK data | C_max,_ (µg/mL), mean (range) | 71.5 (40.0-150) | 55.6(19.8-382) | NM | NM | NM | NM | NM | NM |
|  | T_max_ (days), mean (range) | 27.5 (13.8-30.0) | 23.2 (13.0-28.0) | NM | NM | NM | NM | NM | NM |
|  | AUC_d0-28_ (µg⋅day/mL), mean (range) | 1316 (625-2667) | 964 /9393-4552) | NM | NM | NM | NM | NM | NM |

Note: NR: not recorded; NM: not measured. [a] = terminal elimination half-life and [b] = elimination half-life

**Table S2:** Parameters for adult height, weight, and their coefficient of variations (CVs) used in Sim-North American Hispanic_Latino

| **Parameter** | **Gender** | **Default equation** | **Adjusted equation** |
| --- | --- | --- | --- |
| Height (Equations) | Male | HT = 176.87 + 0.0743*age - 0.0021 * age^2^ | 171.90 + 0.1113*age - 0.0025*age^2^ |
|  | Female | HT = 161.39 + 0.1498*age - 0.0027*age^2^ | 158.24 + 0.1319*age - 0.0027*age^2^ |
| Adult CV for Height (%) | Male | 4.4 | 1.9 |
|  | Female | 4.2 | 1.7 |
| Weight (Equations) | Male | WT = exp(2.7208 + 0.0097*HT) | WT = exp(2.643 + 0.00988*HT) |
|  | Female | WT = (2.936 + 0.008*HT) | WT = exp(2.7383 + 0.0089*HT) |
| Adult CV for Weight (%) | Male | 22.2 | 5.2 |
|  | Female | 26 | 9 |

**Table S3:** PK values by varying f_uGut_

| f_uGut_ | **Mean** | | | |
| --- | --- | --- | --- | --- |
|  | T_Max (d_) | C_Max_ (µg/mL) | AUC_d0-28_ (µg/mL.d) | AUC_d0-¥_ (µg/mL.d) |
| 0.02 | 28 | 24.1 | 476.8 | 783.6 |
| 0.03089 | 28 | 24.1 | 476.8 | 783.5 |
| 0.04771 | 28 | 24.1 | 476.7 | 783.3 |
| 0.07368 | 28 | 24.1 | 476.5 | 783.1 |
| 0.1138 | 28 | 24.1 | 476.3 | 782.7 |
| 0.17575 | 28 | 24.0 | 476.0 | 782.2 |
| 0.27144 | 28 | 24.0 | 475.4 | 781.3 |
| 0.41923 | 28 | 24.0 | 474.6 | 780.0 |
| 0.64748 | 28 | 23.9 | 473.4 | 777.9 |
| 1 | 28 | 23.8 | 471.5 | 774.8 |

**Table S4:** PK cost function as a function of combined C_lint_ and K_p_ in the ASA test

| Run | C_lint_ | K_p_ | Cost function |  | Run | C_lint_ | K_p_ | Cost function |
| --- | --- | --- | --- | --- | --- | --- | --- | --- |
| 1 | 1.18 | 1.00 | 1.954 |  | 51 | 2.55 | 1.00 | 1.475 |
| 2 | 1.18 | 1.38 | 2.014 |  | 52 | 2.55 | 1.38 | 1.421 |
| 3 | 1.18 | 1.89 | 2.071 |  | 53 | 2.55 | 1.89 | 1.366 |
| 4 | 1.18 | 2.60 | 2.117 |  | 54 | 2.55 | 2.60 | 1.316 |
| 5 | 1.18 | 3.58 | 2.145 |  | 55 | 2.55 | 3.58 | 1.270 |
| 6 | 1.18 | 4.92 | 2.139 |  | 56 | 2.55 | 4.92 | 1.232 |
| 7 | 1.18 | 6.77 | 2.088 |  | 57 | 2.55 | 6.77 | 1.203 |
| 8 | 1.18 | 9.31 | 1.985 |  | 58 | 2.55 | 9.31 | 1.189 |
| 9 | 1.18 | 12.80 | 1.836 |  | 59 | 2.55 | 12.80 | 1.196 |
| 10 | 1.18 | 17.60 | 1.658 |  | 60 | 2.55 | 17.60 | 1.230 |
| 11 | 1.38 | 1.00 | 1.439 |  | 61 | 2.97 | 1.00 | 1.709 |
| 12 | 1.38 | 1.38 | 1.498 |  | 62 | 2.97 | 1.38 | 1.656 |
| 13 | 1.38 | 1.89 | 1.556 |  | 63 | 2.97 | 1.89 | 1.603 |
| 14 | 1.38 | 2.60 | 1.607 |  | 64 | 2.97 | 2.60 | 1.553 |
| 15 | 1.38 | 3.58 | 1.644 |  | 65 | 2.97 | 3.58 | 1.509 |
| 16 | 1.38 | 4.92 | 1.659 |  | 66 | 2.97 | 4.92 | 1.470 |
| 17 | 1.38 | 6.77 | 1.639 |  | 67 | 2.97 | 6.77 | 1.440 |
| 18 | 1.38 | 9.31 | 1.573 |  | 68 | 2.97 | 9.31 | 1.420 |
| 19 | 1.38 | 12.80 | 1.464 |  | 69 | 2.97 | 12.80 | 1.415 |
| 20 | 1.38 | 17.60 | 1.321 |  | 70 | 2.97 | 17.60 | 1.432 |
| 21 | 1.61 | 1.00 | 0.998 |  | 71 | 3.47 | 1.00 | 1.907 |
| 22 | 1.61 | 1.38 | 1.056 |  | 72 | 3.47 | 1.38 | 1.856 |
| 23 | 1.61 | 1.89 | 1.114 |  | 73 | 3.47 | 1.89 | 1.805 |
| 24 | 1.61 | 2.60 | 1.166 |  | 74 | 3.47 | 2.60 | 1.756 |
| 25 | 1.61 | 3.58 | 1.209 |  | 75 | 3.47 | 3.58 | 1.713 |
| 26 | 1.61 | 4.92 | 1.236 |  | 76 | 3.47 | 4.92 | 1.675 |
| 27 | 1.61 | 6.77 | 1.238 |  | 77 | 3.47 | 6.77 | 1.643 |
| 28 | 1.61 | 9.31 | 1.203 |  | 78 | 3.47 | 9.31 | 1.620 |
| 29 | 1.61 | 12.80 | 1.127 |  | 79 | 3.47 | 12.80 | 1.609 |
| 30 | 1.61 | 17.60 | 1.103 |  | 80 | 3.47 | 17.60 | 1.613 |
| 31 | 1.87 | 1.00 | 0.879 |  | 81 | 4.05 | 1.00 | 2.074 |
| 32 | 1.87 | 1.38 | 0.822 |  | 82 | 4.05 | 1.38 | 2.025 |
| 33 | 1.87 | 1.89 | 0.765 |  | 83 | 4.05 | 1.89 | 1.977 |
| 34 | 1.87 | 2.60 | 0.787 |  | 84 | 4.05 | 2.60 | 1.930 |
| 35 | 1.87 | 3.58 | 0.832 |  | 85 | 4.05 | 3.58 | 1.887 |
| 36 | 1.87 | 4.92 | 0.866 |  | 86 | 4.05 | 4.92 | 1.850 |
| 37 | 1.87 | 6.77 | 0.882 |  | 87 | 4.05 | 6.77 | 1.819 |
| 38 | 1.87 | 9.31 | 0.870 |  | 88 | 4.05 | 9.31 | 1.794 |
| 39 | 1.87 | 12.80 | 0.897 |  | 89 | 4.05 | 12.80 | 1.778 |
| 40 | 1.87 | 17.60 | 0.994 |  | 90 | 4.05 | 17.60 | 1.775 |
| 41 | 2.19 | 1.00 | 1.201 |  | 91 | 4.72 | 1.00 | 2.214 |
| 42 | 2.19 | 1.38 | 1.145 |  | 92 | 4.72 | 1.38 | 2.169 |
| 43 | 2.19 | 1.89 | 1.089 |  | 93 | 4.72 | 1.89 | 2.122 |
| 44 | 2.19 | 2.60 | 1.038 |  | 94 | 4.72 | 2.60 | 2.077 |
| 45 | 2.19 | 3.58 | 0.992 |  | 95 | 4.72 | 3.58 | 2.035 |
| 46 | 2.19 | 4.92 | 0.955 |  | 96 | 4.72 | 4.92 | 1.999 |
| 47 | 2.19 | 6.77 | 0.930 |  | 97 | 4.72 | 6.77 | 1.969 |
| 48 | 2.19 | 9.31 | 0.927 |  | 98 | 4.72 | 9.31 | 1.944 |
| 49 | 2.19 | 12.80 | 0.951 |  | 99 | 4.72 | 12.80 | 1.926 |
| 50 | 2.19 | 17.60 | 1.006 |  | 100 | 4.72 | 17.60 | 1.917 |

**Table S5:** PK values of based on the selected combinations from the ASA test.

| CL_int_ | K_p_ | AUC_d0-28_ (µg/mL.d) | AUC_d0-∞_ (µg/mL.d) | T_max_ | C_max_ (µg/mL) | Cost Function | Cure rate (%) |
| --- | --- | --- | --- | --- | --- | --- | --- |
| 1.87 | 1 | 981.42 | 1053.29 | 28 | 29.61 | 1.12 | 89.09 |
| 1.87 | 1.37 | 985.61 | 1077.22 | 28 | 38.38 | 1.17 | 89.54 |
| 1.87 | 1.89 | 981.59 | 1100.23 | 28 | 39.09 | 1.22 | 90.45 |
| 1.87 | 2.60 | 965.79 | 1122.15 | 28 | 39.62 | 1.26 | 90.45 |
| 1.87 | 3.57 | 934.66 | 1141.83 | 28 | 39.81 | 1.29 | 91.36 |
| 1.87 | 4.92 | 886.11 | 1158.62 | 28 | 39.44 | 1.30 | 91.82 |
| 1.87 | 6.76 | 819.15 | 1172.58 | 28 | 38.3 | 1.28 | 89.09 |
| 1.87 | 9.30 | 736.54 | 1183.64 | 28 | 36.23 | 1.23 | 83.18 |
| 1.87 | 12.79 | 642.18 | 1191.81 | 28 | 33.19 | 1.14 | 71.81 |
| 1.87 | 17.6 | 543.52 | 1195.95 | 28 | 29.38 | 1.18 | 49.54 |

**Table S6:** ASA tests to determine the optimal activation constant α

| α | AUCd_0-28_ (µg/mL.d) | T_max_ | C_max_ (µg/mL) | Cost Function d_0-28_ |
| --- | --- | --- | --- | --- |
| 0.1 | 195.1 | 28 | 8.2 | 1.7 |
| 0.2 | 390.3 | 28 | 16.4 | 1.4 |
| 0.3 | 585.5 | 28 | 24.6 | 1.2 |
| 0.4 | 780.7 | 28 | 32.8 | 0.9 |
| 0.5 | 975.9 | 28 | 41.0 | 0.6 |
| 0.6 | 1171.1 | 28 | 49.2 | 0.4 |
| 0.7 | 1366.3 | 28 | 57.4 | 0.2 |
| 0.8 | 1561.5 | 28 | 65.6 | 0.2 |
| 0.9 | 1756.7 | 28 | 73.8 | 0.3 |
| 1 | 1951.9 | 28 | 82.0 | 0.6 |
| 1.1 | 2147.0 | 28 | 90.2 | 0.8 |
| 1.2 | 2342.2 | 28 | 98.4 | 1.1 |
| 1.3 | 2537.4 | 28 | 106.6 | 1.4 |
| 1.4 | 2732.6 | 28 | 114.8 | 1.6 |
| 1.5 | 2927.8 | 28 | 123.0 | 1.9 |
| 1.6 | 3123.0 | 28 | 131.2 | 2.2 |
| 1.7 | 3318.2 | 28 | 139.4 | 2.4 |
| 1.8 | 3513.4 | 28 | 147.6 | 2.7 |
| 1.9 | 3708.6 | 28 | 155.8 | 2.9 |
| 2 | 3903.8 | 28 | 164.0 | 3.2 |

**Table S7:** Adult height, weight and their CVs used in the simulations of Sim-NEurCaucasian, Sim-North American Asian and Sim-North American African-American virtual populations.

| Parameter | Gender | Sim-NEurCaucasian | Sim-North American Asian | Sim-North American African-American |
| --- | --- | --- | --- | --- |
| Height (HT) | Male | 183.97 + 0.0743 * age - 0.0021 * age^2^ | 171.07 + 0.0743 * age - 0.0021 * age^2^ | 172.57 + 0.0743 * age - 0.0021 * age^2^ |
|  | Female | 168.49 + 0.1498 * age - 0.0027 * age^2^ | 155.59 + 0.1498 * age - 0.0027 * age^2^ | 157.09 + 0.1498 * age - 0.0027 * age^2^ |
| Adult CV for Height (%) | Male | 0.55 | 1.2 | 1.2 |
|  | Female | 0.6 | 1 | 1 |
| Weight (WT) | Male | Exp(2.6608 + 0.0097 * HT) | Exp(2.3408 + 0.0097 * HT) | Exp(2.5205 + 0.0097 * HT) |
|  | Female | Exp(2.876 + 0.008 * HT) | Exp(2.556 + 0.008 * HT) | Exp(2.733 + 0.008 * HT) |
| Adult CV for Weight (%) | Male | 0.5 | 0.4 | 0.4 |
|  | Female | 0.9 | 1.3 | 1.3 |
| Intracellular PK data | C_max, IC_ (µg/mL), mean (range) | 59.4 (12.3-152.2) | 56.7 (6.2-221.2) | 69.6 (13.7-184.6) |
|  | T_max, IC_ (days), mean (range) | 28 | 28 | 28 |
|  | AUC_d0-29, IC_ (µg⋅day/mL), mean (range) | 1320.1 (336.1-2790.9) | 1273.1 90 (329.0-3258.8) | 1600.9 (373.9-3543.9) |

**Table S8:** Paediatric height, weight and their CVs used in the simulations

| Parameter | Gender | Colombian paediatrics | East African paediatrics |
| --- | --- | --- | --- |
| Height (HT) | Male | 0.0000176179*age^7^ - 0.00119874*age^6^ + 0.0323848*age^5^ - 0.444112*age^4^ + 3.2946*age^3^ - 13.2191*age^2^ + 33.75*age + 58.82152 | 0.0000176179*age^7^ - 0.00119874*age^6^ + 0.0323848*age^5^ - 0.444112*age^4^ + 3.2946*age^3^ - 13.2191*age^2^ + 33.75*age + 52.62152 |
|  | Female | -0.00000151027*age^8^  + 0.000121261*age^7^ -0.0040023 *age^6^  + 0.070179*age^5^ - 0.708233*age^4^ + 4.1872*age^3^ -14.3393*age^2^ + 33.84778*age + 57.735477 | -0.00000151027*age^8^ + 0.000121261*age^7^ -0.0040023*age^6^ + 0.070179*age^5^ - 0.708233*age^4^ + 4.1872*age^3^ -14.3393*age^2^ + 33.84778*age + 51.535477 |
| Adult CV for Height (%) | Male | 1.5 | 1.7 |
|  | Female | 1 | 1.2 |
| Weight (WT) | Male | 7.826 * (1.0 - exp(-1.2*age)) + exp((HT * 0.0209) + (0.023 * age)) | 2.626 * (1.0 - exp(-1.2*age)) + exp((HT * 0.0209) + (0.023 * age)) |
|  | Female | 5.454 * (1.0 - exp(- 1.57*age)) + exp((HT * 0.0224) + (0.019 * age)) | 0.254 * (1.0 - exp(-1.57*age)) + exp((HT * 0.0224) + (0.019 * age)) |
| Adult CV for Weight (%) | Male | 15 | 18 |
|  | Female | 10 | 13 |

**Table S9:** Miltefosine PK values at different doses for adult and paediatric populations

| Parameters | | Sim-North American Hispanic-Latino | Sim-North American Asian | Sim-Paediatric |
| --- | --- | --- | --- | --- |
| Total number of patients | | 22 x 10 trials | 30 x 10 trials | 22 x 10 trials |
| Demographic data | Female patients, n(%) | 50 | 50 | 50 |
|  | Age (years) | 41.8 (18-65) | 41.3 (18-65) | 7 (2-12) |
|  | Body weight (Kg), mean (range) | 80.8 (40-174 | 68.7 (39-144) | 24.3 (8.7-56.5) |
|  | Height (cm) | 163.6 (141-193) | 163 (141-192) | 120.7 (86.8-152.2) |
| PK data at 2 mg/kg | Cure rate (%) | 78.6 | 77.7 | 48.6 |
|  | C_max, PL_ (µg/mL), mean (range) | 34.2 (11.8-80.8) | 32.1 (10.2-73.5) | 22.4 (6.5-64.3) |
|  | T_max, PL_ (days), mean (range) | 28 | 28 | 28 |
|  | t_½, PL_ (days), mean (range) | 5.1 (4.6-6.4) | 5.0 (4.6-5.6) | 4.6 (4.0-5.8) |
|  | AUC_d0-28, PL_ (µg⋅day/mL), mean (range) | 732.0 (313.4-1531.5) | 719.7 (271.2-1417.1) | 535.5 (176.9-1277.9) |
|  | AUC_d0-∞, PL_ (µg⋅day/mL), mean (range) | 1012.6 (332.6-2901.4) | 945 (285.8-2501.2) | 637.2 (184.6-2076.7) |
| PK data at 2.5 mg/kg | Cure rate (%) | 95 | 92.7 | 70.5 |
|  | C_max, PL_ (µg/mL), mean (range) | 42.8 (14.8-101) | 40.2 (12.7-91.9) | 28 (8.2-80.4) |
|  | T_max, PL_ (days), mean (range) | 28 | 28 | 28 |
|  | t_½, PL_ (days), mean (range) | 5.1 (4.6-6.4) | 5.0 (4.6-5.6) | 4.6 (4.0-5.8) |
|  | AUC_d0-28, PL_ (µg⋅day/mL), mean (range) | 952.7 (391.7-1914.1) | 899.6 (339.1-1771.4) | 669.4 (221.1-1597.4) |
|  | AUC_d0-∞, PL_ (µg⋅day/mL), mean (range) | 1265.8 (415.8-3626.8) | 1181.3 (357.2-3126.5) | 796.5 (230.7-2595.9) |
| PK data at 3 mg/kg | Cure rate (%) | 98.6 | 97.4 | 81.9 |
|  | C_max, PL_ (µg/mL), mean (range) | 51.4 (17.8-121.2) | 48.2 (15.3-110.3) | 33.6 (9.8-96.5) |
|  | T_max, PL_ (days), mean (range) | 28 | 28 | 28 |
|  | t_½, PL_ (days), mean (range) | 5.1 (4.6-6.4) | 5.0 (4.6-5.5) | 4.6 (4.0-5.8) |
|  | AUC_d0-28, PL_ (µg⋅day/mL), mean (range) | 1143.3(470.1-2297.2) | 1079.6 (406.9-2125.7) | 803.2 (265.4-1916.9) |
|  | AUC_d0-∞, PL_ (µg⋅day/mL), mean (range) | 1519 (498.9-4352.1) | 1417.6(428.7-3751.8) | 955.8 (276.9-3115) |
| PK data at 3.5 mg/kg | Cure rate (%) | 100 | 99.34 | 89.1 |
|  | C_max, PL_ (µg/mL), mean (range) | 60 (20.7-141.4) | 56.3 (17.8-128.7) | 39.2 (11.5-112.6) |
|  | T_max, PL_ (days), mean (range) | 28 | 28 | 28 |
|  | t_½, PL_ (days), mean (range) | 5.1 (4.6-6.4) | 5.0 (4.6-5.5) | 4.6 (4.0-5.8) |
|  | AUC_d0-28, PL_ (µg⋅day/mL), mean (range) | 1333.9 (548.4-2680.1) | 1259.5 (474.7-2480) | 937.1 (309.6-2236.4) |
|  | AUC_d0-∞, PL_ (µg⋅day/mL), mean (range) | 1772.2 (582.1-5077.5) | 1653.9 (500.1-4377.1) | 1115.1 (323-3634.2) |

Table S10: Pharmacokinetic Parameters of Miltefosine in Paediatrics at a dose of 3.9 mgl/kg

| Parameters | | Sim-Paediatric |
| --- | --- | --- |
| Total number of patients | | 22 x 10 trials |
| Demographic data | Female patients, n(%) | 50 |
|  | Age (years) | 7 (2-12) |
|  | Body weight (Kg), mean (range) | 24.3 (8.7-56.5) |
|  | Height (cm) | 120.7 (86.8-152.2) |
| PK data at 3.9 mg/kg | Cure rate (%) | 91.8 |
|  | C_max, PL_ (µg/mL), mean (range) | 43.7(12.8-125.4) |
|  | T_max, PL_ (days), mean (range) | 28 |
|  | t_½, PL_ (days), mean (range) | 3.5 (3.1-3.6) |
|  | AUC_d0-28, PL_ (µg⋅day/mL), mean (range) | 1044.2 (345.0-2492.0) |
|  | AUC_d0-∞, PL_ (µg⋅day/mL), mean (range) | 1242.5 (360.0-4049.6) |

Table S11: Predictions of the fraction of dose absorbed and oral bioavailability by the optimised PBPK model developed for different populations

| Parameters | Adults | | | | Paediatrics | |
| --- | --- | --- | --- | --- | --- | --- |
|  | Colombia | Europeans | Nepalese | East Africans | Colombian | East African |
| Bioavailability (F) | 0.78 | 0.81 | 0.78 | 0.78 | 0.84 | 0.83 |
| Fraction absorbed (fa) | 0.75 | 0.78 | 0.75 | 0.75 | 0.77 | 0.74 |

**Figure S1:** Prediction results at different combinations of CL_int_ and K_p_ selected from the ASA test: (a) plasma exposure profiles; (b) AUC_d0-28_ distributions

| 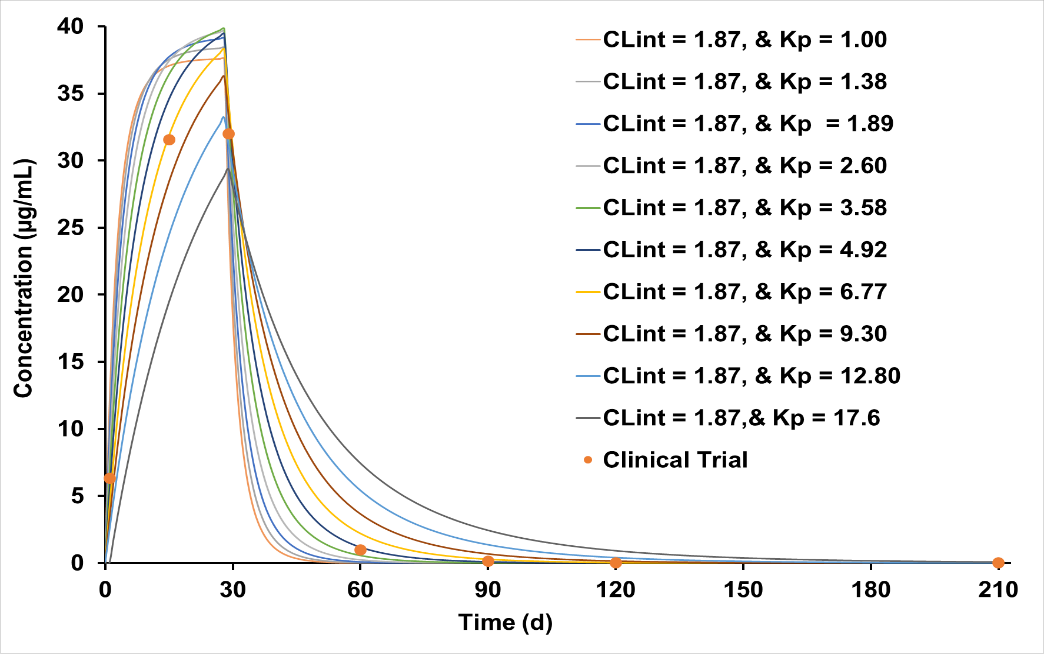(a) |
| --- |
| 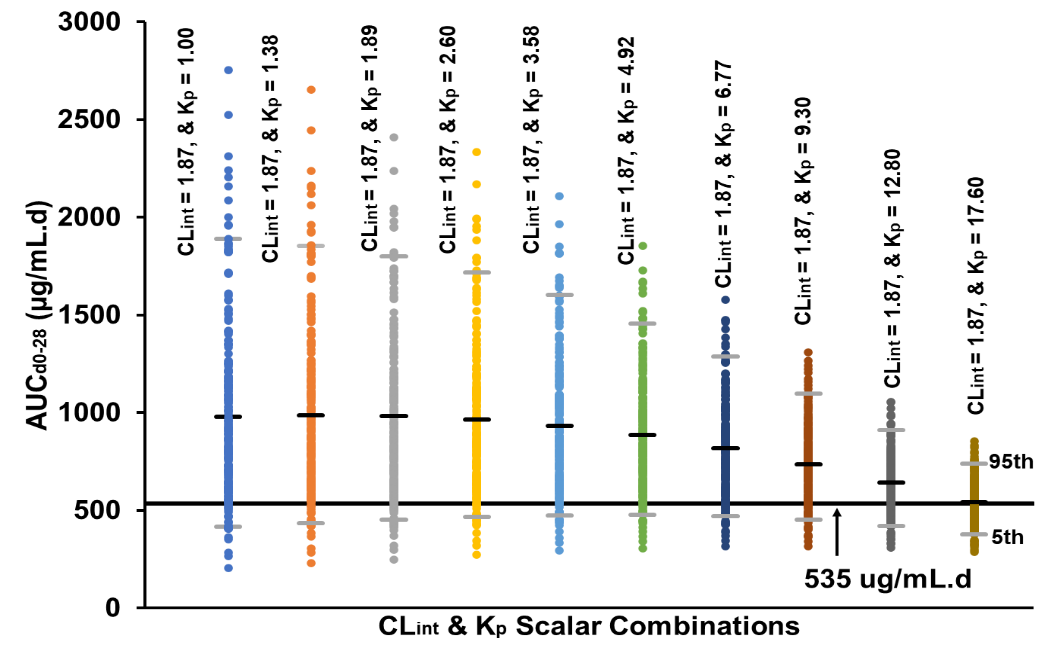(b) |

**Figure S2:** Miltefosine exposure profiles in PBMCs at different activation constants α


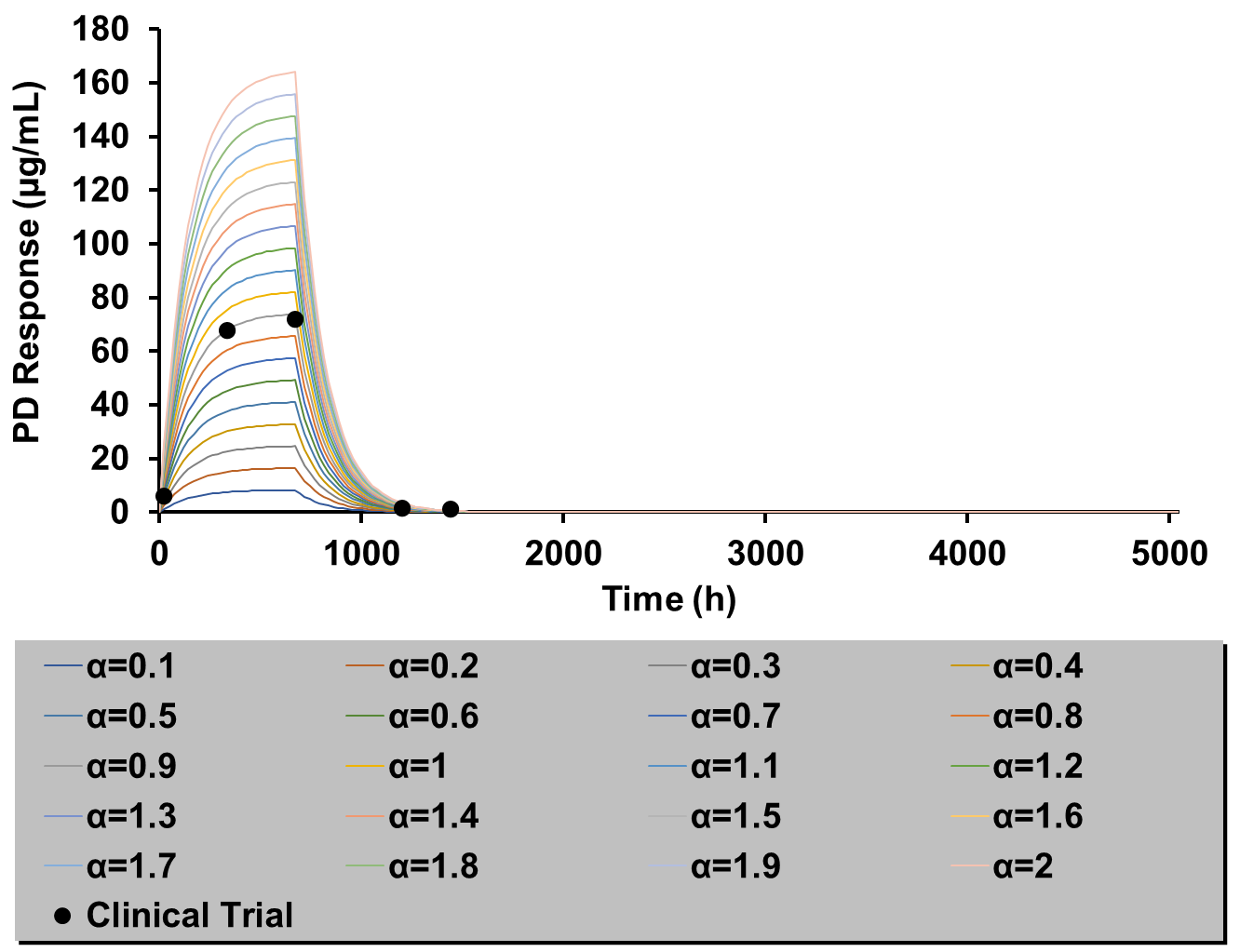


**Figure S3:** Miltefosine exposure profiles in PBMCs of the adult simulations: (a) Dutch military personnel; (b) Nepalese adults; (c) East African adults

| 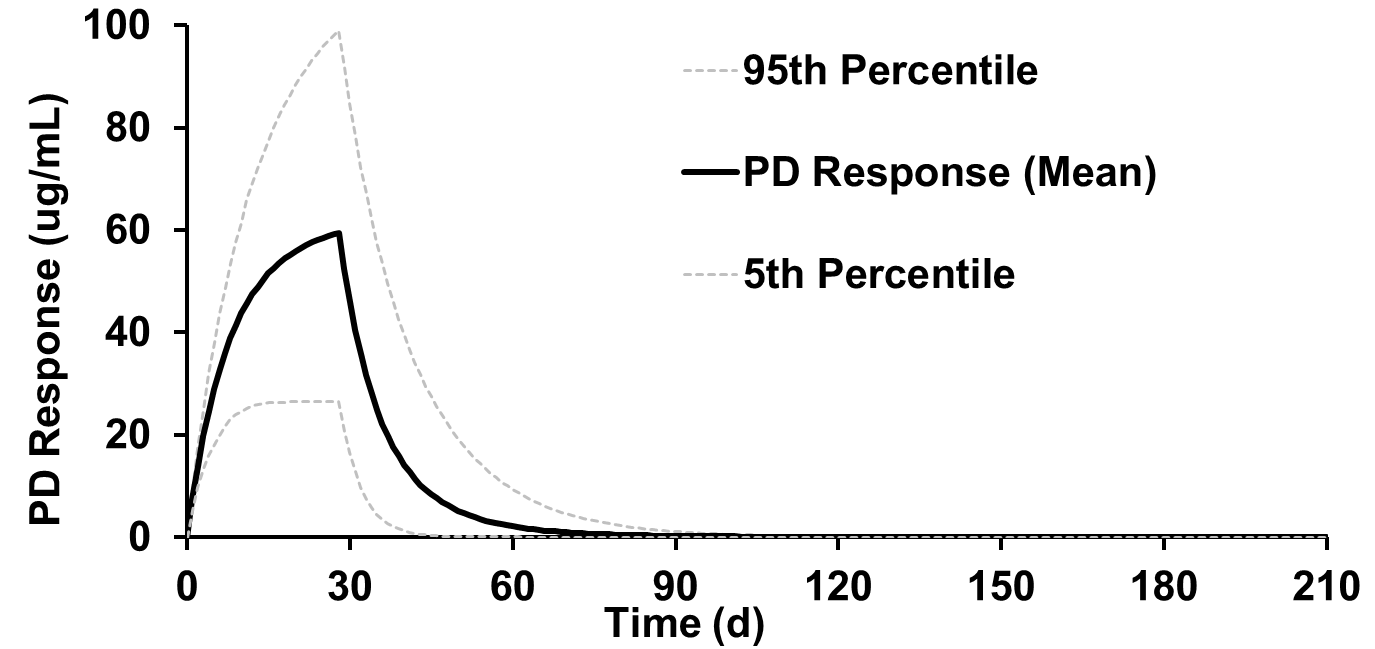  (a) | 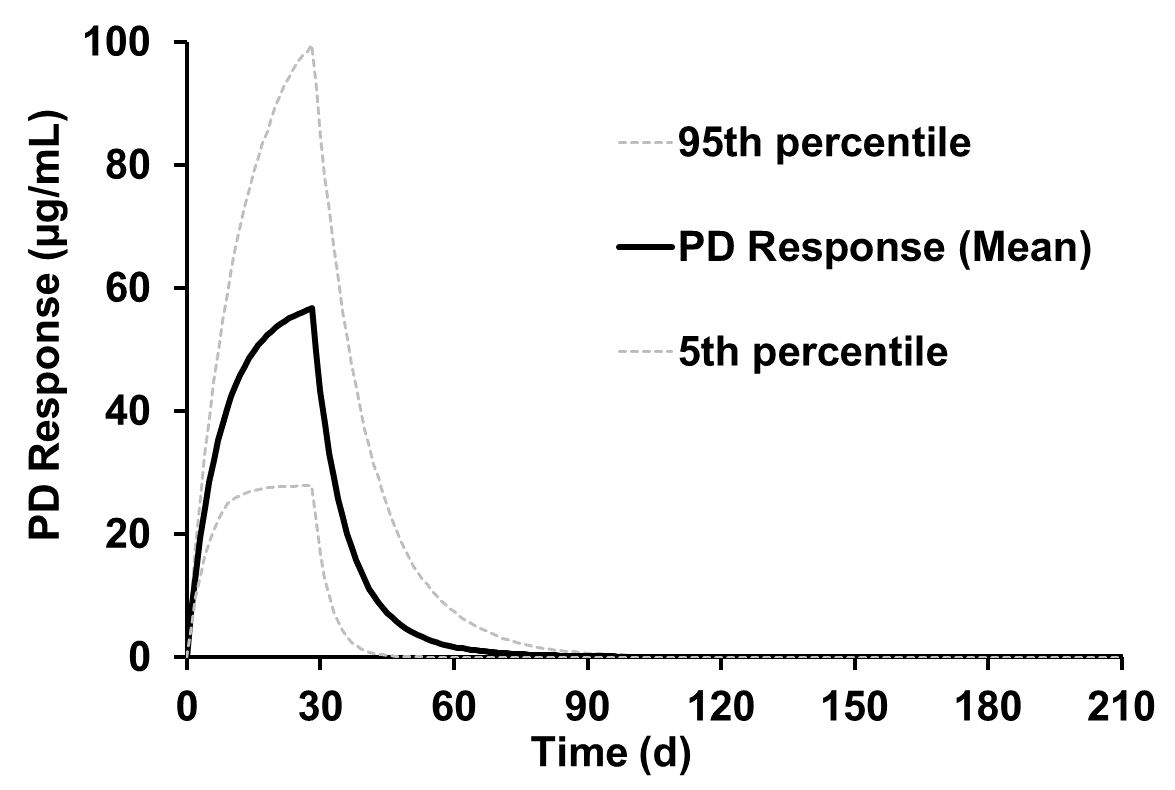  (b) |
| --- | --- |
| 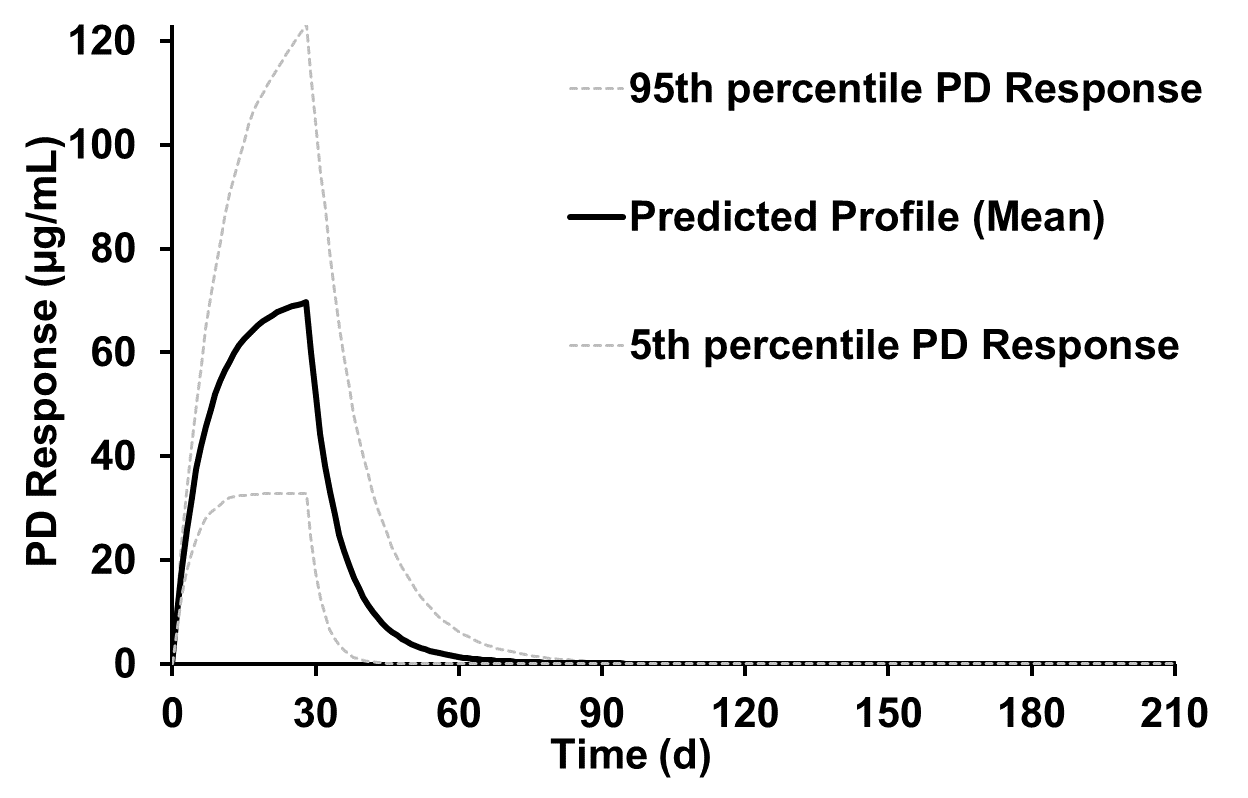  (c) |  |

Figure S4: Miltefosine exposure profiles in plasma and PBMCs of different population under different doses: (a) Sim-North American Hispanic_Latino adults; (b) Sim-North American Asian adults; (c) Sim-Paediatrics

|  | Plasma | PBMCs |
| --- | --- | --- |
| (a) | 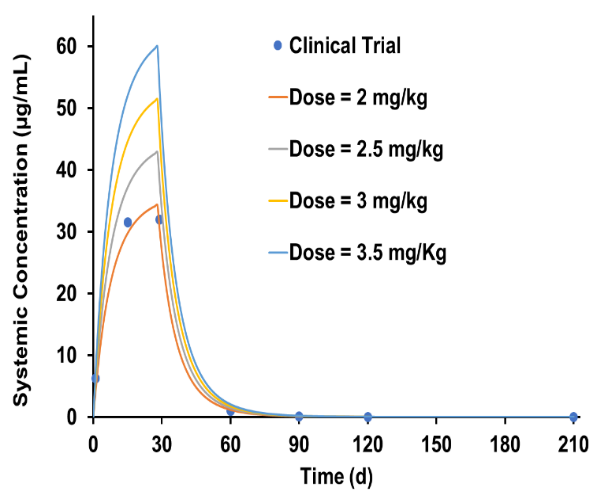 | **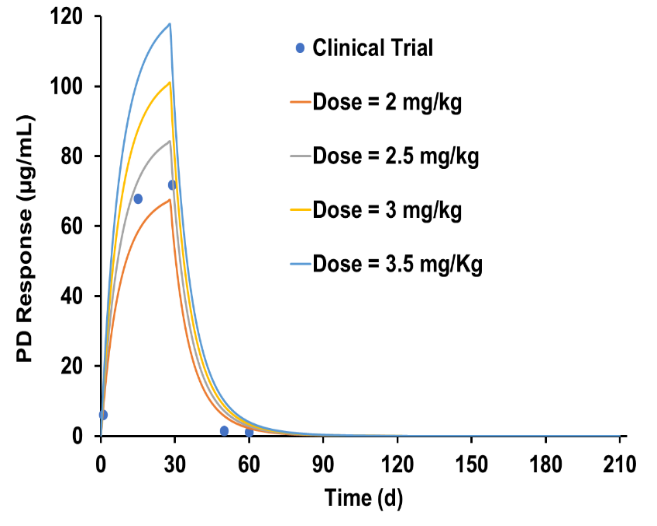** |
| (b) | **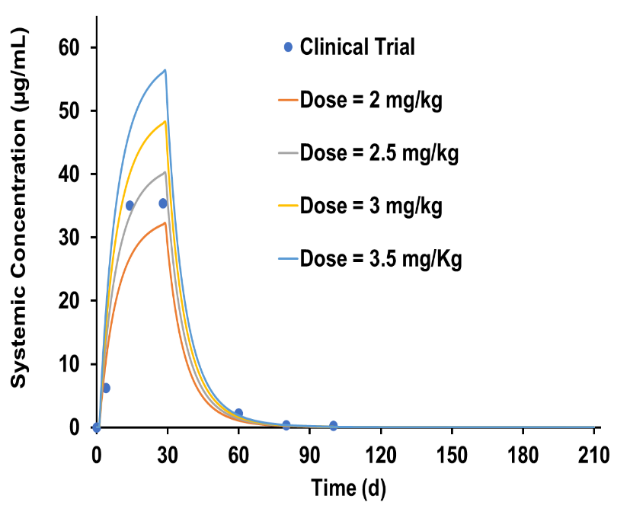** | **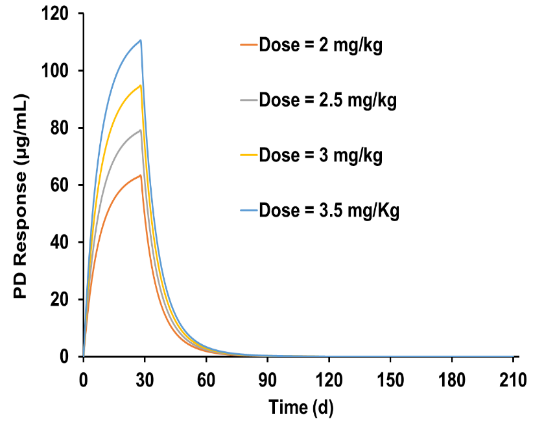** |
| (c) | 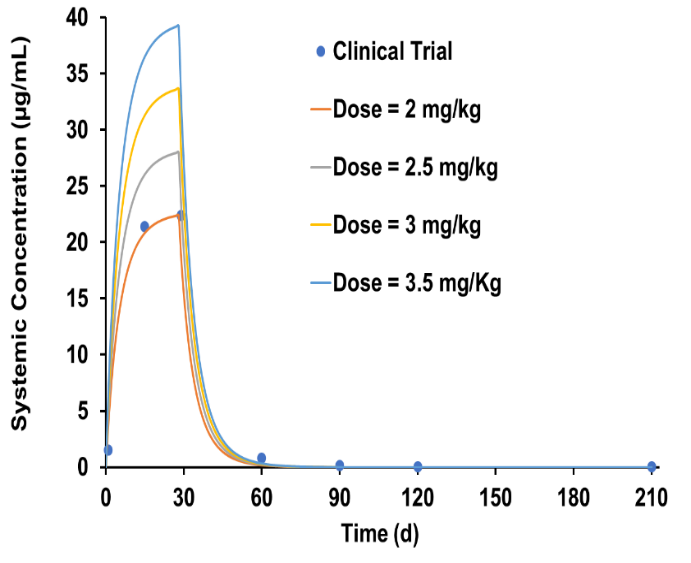 | **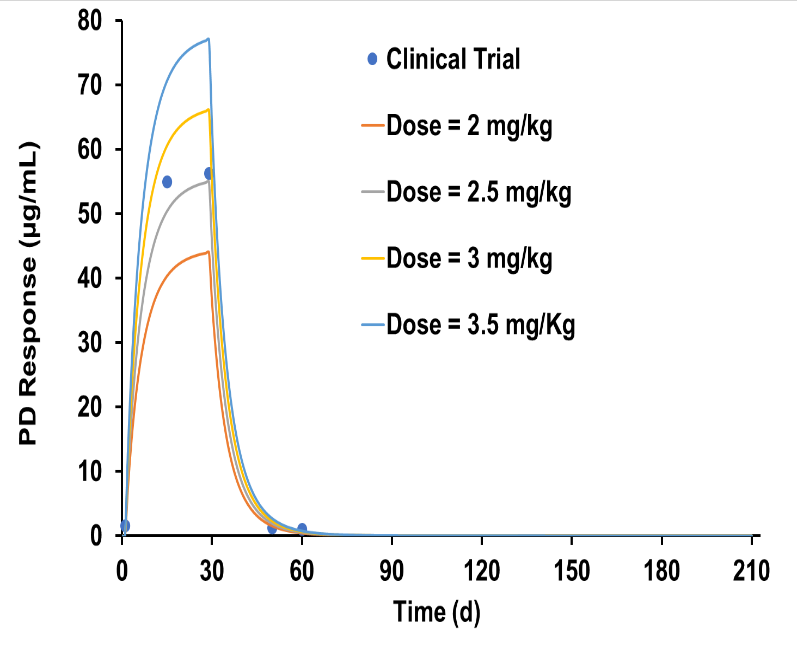** |

Figure S5: Miltefosine exposure profiles in plasma and PBMCs in Sim-Paediatric population using allometric dosing: (a) Plasma (b) PBMC and (c) Cure rate

| 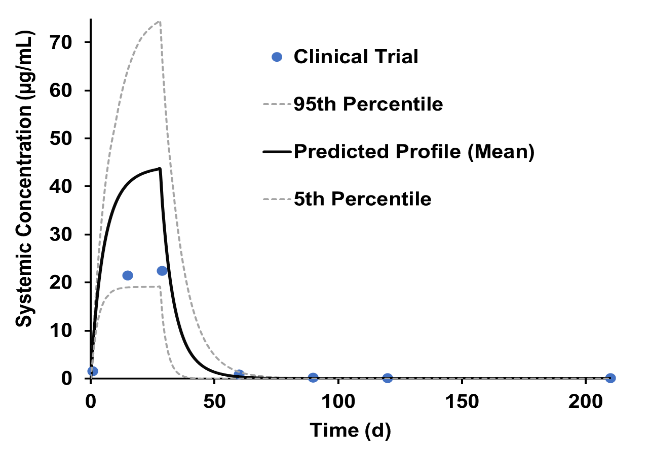(a) | 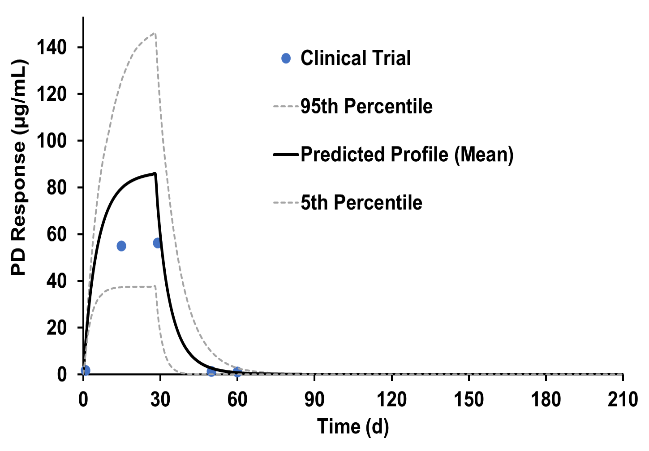(b) |
| --- | --- |
| 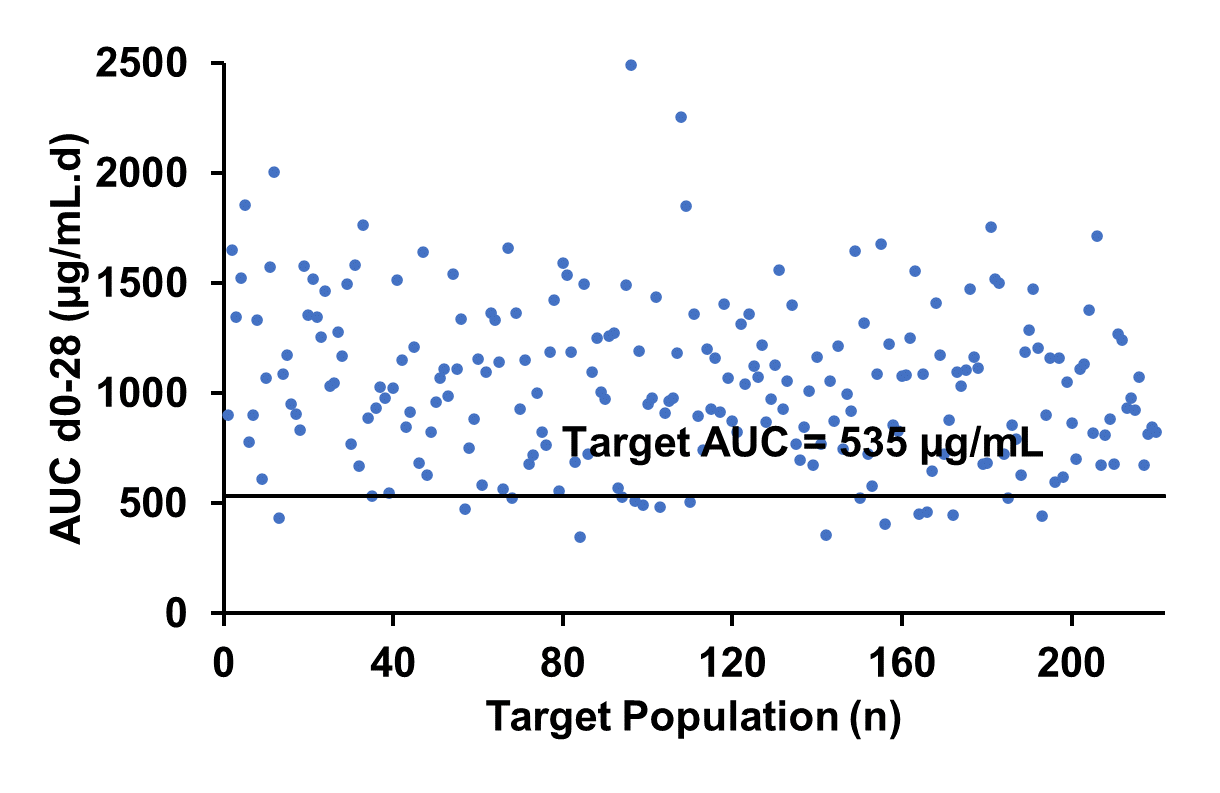(c) |  |

1. * corresponding author: [mli@dmu.ac.uk](mailto:mli@dmu.ac.uk) [↑](#footnote-ref-1)
